# Significance of miR-196b in Tumor-Related Epilepsy of Patients with Gliomas

**DOI:** 10.1371/journal.pone.0046218

**Published:** 2012-09-25

**Authors:** Gan You, Wei Yan, Wei Zhang, Yongzhi Wang, Zhaoshi Bao, Shouwei Li, Shaowu Li, Guilin Li, Yijun Song, Chunsheng Kang, Tao Jiang

**Affiliations:** 1 Department of Neurosurgery, Beijing Tiantan Hospital, Capital Medical University, Beijing, China; 2 Department of Neurosurgery, Tianjin Medical University General Hospital, Laboratory of Neuro-Oncology, Tianjin Neurological Institute, Tianjin, China; 3 Department of Neurosurgery, Beijing Sanbo Brain Hospital, Capital Medical University, Beijing, China; 4 Department of Neuroradiology, Beijing Tiantan Hospital, Capital Medical University, Beijing, China; 5 Department of Neuropathology, Beijing Neurosurgical Institute, Beijing, China; Sun Yat-sen University Medical School, China

## Abstract

**Objectives:**

Seizure is a common presenting symptom of primary brain tumors. There are no published studies regarding the roles of MicroRNA (miRNA) in tumor-related epilepsy. The authors set out to correlate miR-196b expression in low-grade glioma patients with pre-operative seizures and post-operative seizure control.

**Methods:**

Twenty-three patients with WHO grade II astrocytomas and 83 similar patients for independent validation were included. Follow-up visits regarding seizure prognosis were scheduled at 6 months. MiRNA profiling was used to identify differentially expressed miRNAs. The most important miRNA was determined by quantitative reverse-transcriptase polymerase chain reaction (q-PCR) in the validation cohort. Gene ontology (GO) analysis and whole genome mRNA profiling was performed to investigate the underlying biological processes.

**Results:**

Array results showed that 30 miRNAs were overexpressed and 10 miRNAs were underexpressed (with more than 2 fold change) in patients with pre-operative seizures. MiR-196b was validated in the independent validation cohort. Patients with good seizure prognosis exhibited low levels of miR-196b expression compared with those who had poor seizure prognosis in the group without pre-operative seizures. Biological processes that relate to transcription and cell cycles were over-represented in the miR-196b-associated gene expression signature. MiR-196b-associated gene expression profiling was characterized by enrichment of genes usually involved in cell proliferation.

**Conclusions:**

We have provided the first evidence that expression of miR-196b was associated with the occurrence of pre-operative seizures in low-grade gliomas, and may predict seizure prognosis in patients without pre-operative seizures. Targeted treatments that decrease endogenous levels of miR-196b might represent novel therapeutic strategies.

## Introduction

Epilepsy is a common presenting symptom of primary brain tumors, particularly low-grade gliomas (LGGs). Most patients with LGGs experience epileptic seizures at disease onset [1]–[5], but not all have seizures, despite having similar histology and tumor location. This suggests that some distinct molecular genetic characteristics may exist for tumors causing different clinical symptoms. The presence of seizures may, to some extent, be related to the biological behavior of gliomas. Seizures play an important role in patients' postoperative quality of life [5], especially the patients with LGGs because of the duration of their survival. Although more than half of the patients with LGGs may have favorable seizure prognosis after surgery, about 30% of these patients suffer from uncontrolled seizures in spite of treatment with different antiepileptic drugs (AEDs) [2], [4].

MicroRNAs (miRNAs) are single-stranded non-coding RNAs recognized as endogenous regulators of post-transcriptional gene expression. These small RNAs are capable of controlling gene expression by mediating either mRNA degradation or translation inhibition [6]. Several miRNAs have been found in the human brain, and they play a crucial role in a wide range of biological processes, such as regulation of the innate and adaptive immune response [7], [8]. MiRNAs are implicated in maintaining the cell fate of immune cells (e.g. miR-181a/223), and they are involved in innate immunity by regulating Toll-like receptor signaling and ensuing cytokine response (e.g. miR-146). Furthermore, miRNAs regulate central elements of the adaptive immune response such as antigen presentation (e.g. miR-155). Increasing evidence highlights the functions of miRNAs that participate in the underlying molecular mechanisms of neurological diseases, such as Parkinson's disease and Alzheimer's disease [9], [10]. These small non-coding RNAs may provide opportunities for diagnosis and treatment of a damaged nervous system. There are numerous reports supporting the involvement of inflammatory and immune processes in temporal lobe epilepsy (TLE) [11], [12]. However, there are no published studies regarding the roles of miRNAs in tumor-related epilepsy, a major neurological disorder affecting nearly ten million people worldwide.

Previous studies have indicated that miR-196b may be a prognostic predictor in glioblastoma (GBM) patients [13], [14]. Here, we report the expression pattern of miR-196b using the expression values from microarrays of 23 astrocytoma tissues. This result was validated in an independent cohort containing 83 samples. Subsequently, a miR-196b-associated gene-expression profile was characterized by some enrichment of genes related to transcription and cell cycles.

## Methods

### Patients and tissue samples

**Figure 1:**
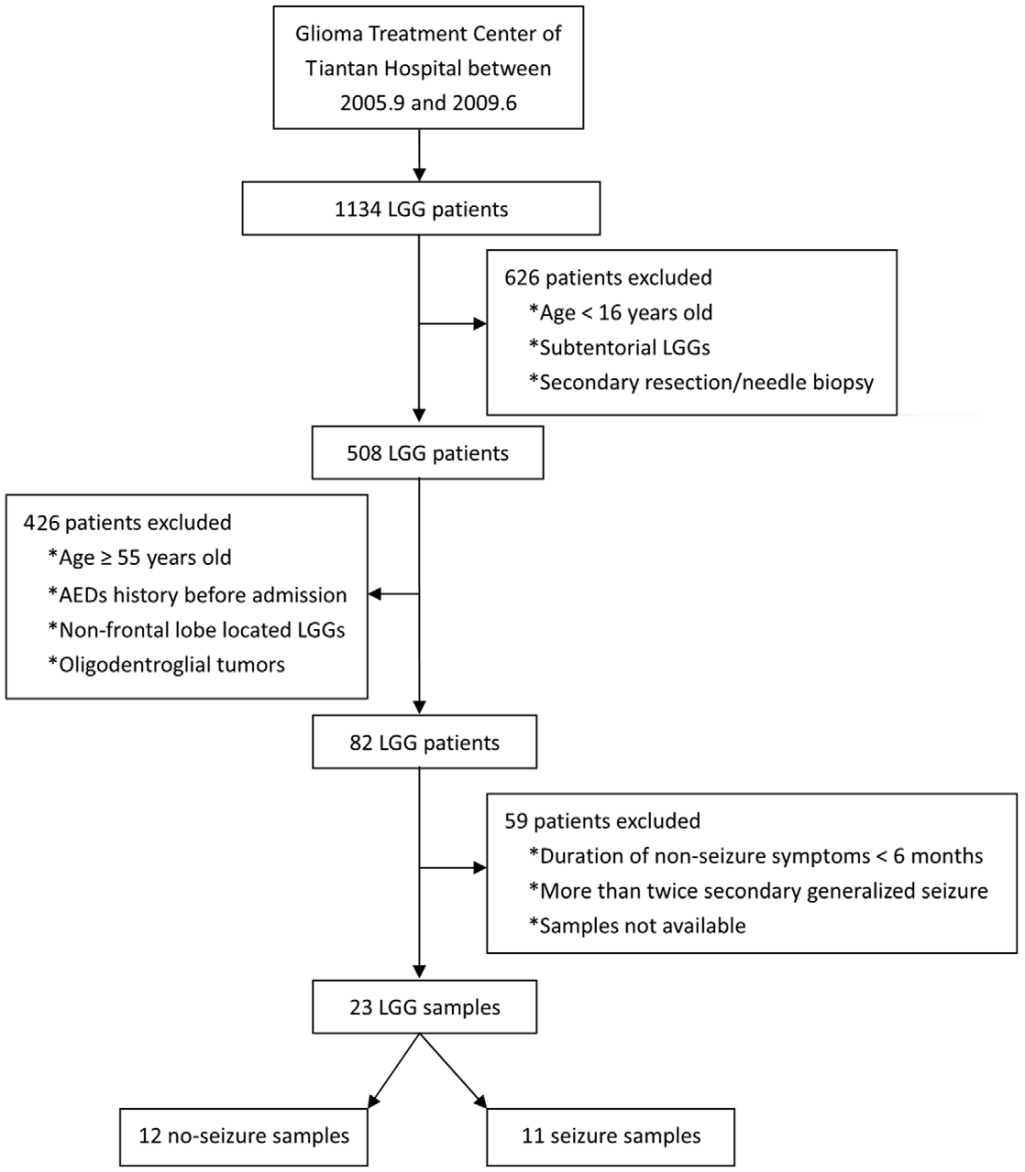
Procedure for the selection of 23 samples used for whole genome microRNA profiling.

This study was approved by the Ethics Committee of Beijing Tiantan Hospital and written informed consent was obtained from all the patients included. Between September 2005 and June 2009, 1134 patients with LGGs were treated at the Glioma Treatment Center of Tiantan Hospital (Beijing, China). Of these patients, 508 (>16-years-old) underwent primary resection of supratentorial LGGs. All patients were pathologically confirmed and diagnosed as having WHO Grade II gliomas, including 48 oligodendrogliomas (O), 229 astrocytomas (A) and 231 oligoastrocytomas (OA). Tissue samples were recovered immediately after surgery, snap-frozen using liquid nitrogen vapors and stored at −80°C until needed. For the purpose of creating more homogeneous sample conditions, only samples that strictly met the following criteria were selected: 1) age <55-years-old; 2) no medication involving AEDs before admission; 3) a single lesion mainly focused at the frontal lobe as determined by MRI; and 4) pure WHO grade II astrocytomas by histopathology. We excluded older patients (≥55-years-old), patients on AEDs medication before admission, patients with partial or occipital lobe or 3 lobes of tumor location, as well as those with secondary resection or needle biopsy only. To make a comparison between seizure and non-seizure tissue, we defined the 2 groups as the seizure (patients who had secondary generalized seizure once or twice) and non-seizure (patients who demonstrated non-seizure symptoms for more than 6 months) groups. Twenty-three samples were eventually included for whole genome miRNA microarray analysis (Figure 1). Each of these samples was proved to contain >80% of tumor cells. The clinical features of these cases are summarized in Table 1. Another 83 samples, including O, A, and OA were obtained for validation using quantitative reverse-transcriptase polymerase chain reaction (q-PCR) analysis (Table 2).

**Table 1:** Summary of clinical data.

Patients	Gender/Age (yrs)	Seizures	Duration of disease (days)	Seizure type	Tumor location^a^	Diagnosis
P1	F/37	Yes	6	SGS	FT	II A
P2	M/30	Yes	4	SGS	F	II A
P3^b^	F/30	Yes	6	SGS	F	II A
P4	M/44	Yes	10	SGS	F	II A
P5	M/24	Yes	14	SGS	FT	II A
P6^b^	F/36	Yes	1	SGS	F	II A
P7	F/38	Yes	2	SGS	F	II A
P8	M/49	Yes	7	SGS	F	II A
P9	M/30	Yes	14	SGS	F	II A
P10	M/45	Yes	7	SGS	FT	II A
P11	M/31	Yes	7	SGS	F	II A
P12	F/40	No	1800	–	F	II A
P13	M/54	No	900	–	F	II A
P14^b^	M/37	No	600	–	F	II A
P15^b^	M/37	No	210	–	F	II A
P16	M/47	No	180	–	F	II A
P17	F/52	No	270	–	FT	II A
P18	M/21	No	259	–	F	II A
P19^b^	F/34	No	240	–	F	II A
P20	F/29	No	240	–	F	II A
P21	M/54	No	360	–	FT	II A
P22	M/18	No	360	–	F	II A
P23	M/47	No	570	–	F	II A

a F, frontal; FT, frontal lobe with temporal involvement;

SGS, secondary generalized seizures;

II A, WHO grade II astrocytoma.

b PCR samples used for internal validation.

**Table 2:** Clinical characteristics and univariate analysis of markers predicting pre-operative seizures and seizure control at 6 months after surgery in 83 patients^a^.

Variable	Seizure Group (n = 47)				No-seizure Group (n = 36)				p Value^b^
		Seizure prognosis				Seizure prognosis			
	Total	Engel I	Engel II–IV	p Value	Total	Good	Poor	p Value	
No. of patients	46	32	14	–	32	24	8	–	–
Male sex	22(47.8)	15(46.9)	7(50.0)	0.845	20(62.5)	15(62.5)	5(62.5)	1.000	0.270
Median age in yrs (range)	38.7 (20–61)	37 (20–58)	42 (24–61)	0.125	41.2 (18–56)	40.8 (18–56)	42.3 (20–56)	0.716	0.241
Tumor location (involved)									
Frontal	24(52.2)	16(50.0)	8(57.1)	0.655	15(46.9)	11(45.8)	4(50.0)	0.838	0.923
Temporal	26(56.5)	19(59.4)	7(50.0)	0.555	20(62.5)	16(66.7)	4(50.0)	0.399	0.935
Parietal	1(2.2)	0(0)	1(7.1)	0.126	2(6.3)	2(8.3)	0(0)	0.399	0.407
Insula	6(13.0)	5(15.6)	1(7.1)	0.432	6(18.8)	4(16.7)	2(25.0)	0.601	0.583
Tumor pathology				0.449				0.298	0.729
Astrocytoma	27(58.7)	17(53.1)	10(71.4)		16(50.0)	11(45.8)	5(62.5)		
Oligodendroglioma	3(6.5)	2(6.3)	1(7.1)		5(15.6)	3(12.5)	2(25.0)		
Oligoastrocytoma	16(34.8)	13(40.6)	3(21.4)		11(34.4)	10(41.7)	1(12.5)		
Extent of resection				0.104				0.008^d^	–
Gross-total	18(39.1)	15(46.9)	3(21.4)		17(53.1)	16(66.7)	1(12.5)		
Subtotal	28(60.9)	17(53.1)	11(78.6)		15(46.9)	8(33.3)	7(87.5)		
KPS score≥80	39(88.6)	28(93.3)	11(78.6)	0.151	26(86.7)	18(81.8)	8(100)	0.195	–
Radiotherapy	43(95.6)	29(93.5)	14(100)	0.331	28(96.6)	21(95.5)	7(100)	0.566	–
Chemotherapy	5(11.1)	4(12.9)	1(7.1)	0.569	5(17.2)	4(18.2)	1(14.3)	0.812	–
High miR-196b expression^c^	27(58.7)	19(59.4)	8(57.1)	0.887	16(50)	15(62.5)	1(12.5)	0.014^d^	0.012^d^

a Values represent numbers of patients with percentages in parentheses unless otherwise indicated.

b Comparing patients with no history of seizures to those with a history of seizures.

c Higher than the average ΔCt values.

d Statistically significant.

### Management and evaluation of seizures

Each patient was routinely prescribed sodium Valproate (0.6–1.2 g/day) at admission regardless of their symptoms. After surgery, patients who presented with or without preoperative seizures were discontinued the same AED after 3 months or 2 weeks, respectively, upon demonstration of seizure-free conditions. For outcome analysis, Engel classification was dichotomized as Class I (completely seizure-free) versus Class II–IV (not seizure-free) [2], [15] at 6 and 12 months after surgery. For patients with no history of seizure, more than one episode of un-evoked postoperative seizure meant “not seizure-free”. Early post-operative seizures (within one week after surgery) were not under consideration for evaluating seizure prognosis. Because better follow-up was available for 6 months, seizure control at this time was the primary endpoint.

### RNA preparation

Total RNA from frozen tumor tissues was extracted using the mirVana miRNA Isolation kit (Ambion, Austin, TX) according to the manufacturer's protocol. RNA was quantitated using a ND-1000 UV-VIS Spectrophotometer (NanoDrop Technologies) and the integrity of RNA was assessed with a RNA 6000 Labchip kit in combination with an Agilent 2100 Bioanalyzer (Agilent) according to the manufacturer's instructions. RNA samples used in this study all had an OD 260/280 ratio above 1.9 and an RNA integrity number (RIN) greater than 7.0.

### MicroRNA expression profiling

Total RNA was polyadenylated and then converted to cDNA using a biotin-labeled oligonucleotide dT primer with a universal PCR sequence. After cDNA synthesis, miRNA were individually detected using specific oligonucleotides. A single miRNA-specific oligonucleotide (MSO) was designed against each mature miRNA sequence, and miRNA-specific primers were extended using DNA polymerase. Universal primers were used to amplify the cDNA templates and the primer complimentary to the array was fluorescently labeled. The labeled, single-stranded PCR products were hybridized to a Human v2.0 miRNA Expression BeadChip (Illumina Inc., San Diego, CA) with 1,146 human miRNAs (97% coverage of the miRBase 12.0 database).

### Quantitative RT-PCR

Quantitative RT-PCR was performed using a standard TaqMan PCR kit on an ABI 7900 real-time PCR system (Applied Biosystems, Foster City, CA). All the primers and probes for hsa-miR-196b and U6 rRNA endogenous controls for TaqMan microRNA assays were purchased from Applied Biosystems. Thermal cycling for the quantitative RT-PCR was carried out according to the manufacturer's recommendation and the relative expression levels were calculated using the comparative Ct method.

### Gene Expression Profiling

Microarray analysis was performed using an Agilent Whole Human Genome Array according to the manufacturer's instructions. The integrity of total RNA was checked using an Agilent 2100 Bioanalyzer (Agilent, Santa Clara, USA). The cDNA and biotinylated cRNA were synthesized and hybridized to the array; data were acquired using an Agilent G2565BA Microarray Scanner System and Agilent Feature Extraction Software (v9.1). Probe intensities were normalized using GeneSpring GX 11.0.

### Statistical Analysis

Associations for miR-196b expression, if considered as a continuous variable, with baseline clinical features were analyzed with Student's t-test using GraphPad Prism 4.0. Univariate analyses were carried out using the Chi-square test for dichotomous variables and the Mann-Whitney U-test for continuous non-parametric data. Logistic regression models were constructed to analyze factors related to the probability of post-operative seizure control using SPSS 13.0 for Windows. Gene ontology (GO) analysis was performed using David [16]. A two-sided P-value less than 0.05 was regarded as statistically significant.

## Results

### Association of miR-196b expression with occurrence of pre-operative seizures in LGGs

**Figure 2:**
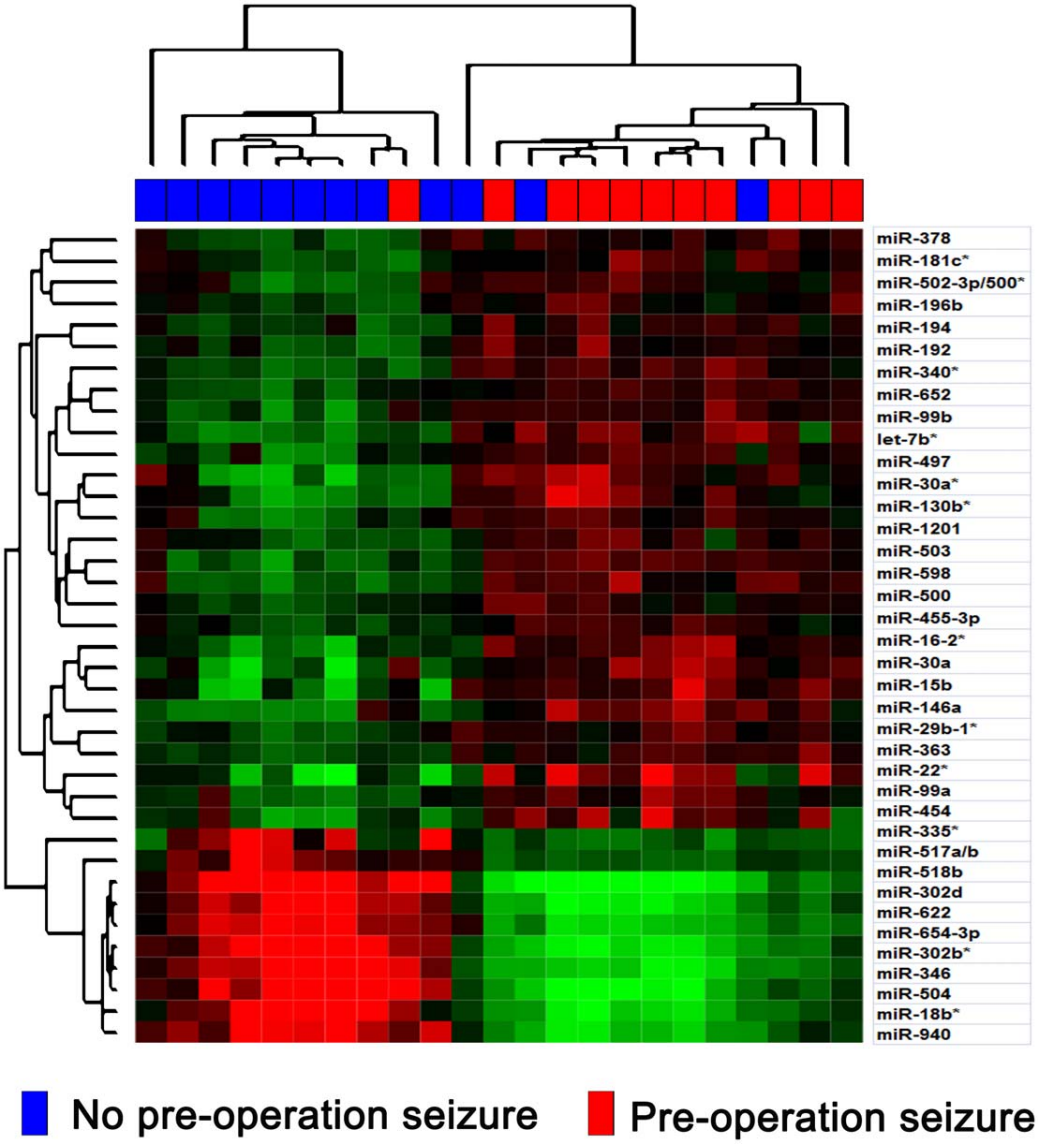
Heat map of the differentially expressed genes identified using SAM analysis (Q value <1%) showing 30 overexpressed and 10 underexpressed miRNAs in seizure group.

**Figure 3:**
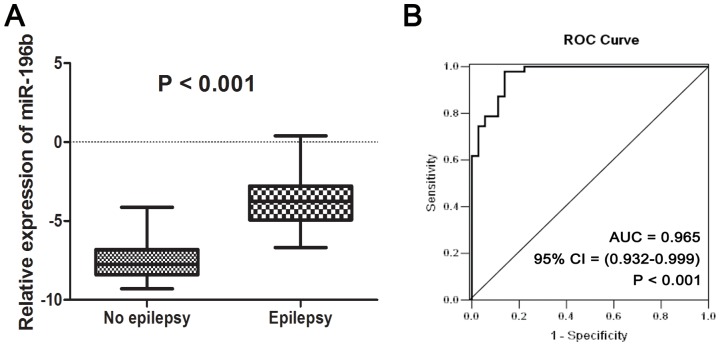
Association of miR-196b expression by qPCR with the occurrence of pre-operative seizures in LGGs. (A) Patients with pre-operative seizures exhibited higher expression levels of miR-196b when compared with those without pre-operative seizures. (B) The diagnostic role of miR-196b for determining the occurrence of pre-operative seizures using receiver operating characteristic curve analysis.

To underscore the differentially expressed miRNAs between patients with and without pre-operative seizures, 23 samples were subjected to whole genome miRNA profiling (Figure 1). The differentially expressed genes were identified using SAM analysis. Using a criterion of p<0.01 and at least 2-fold change, 30 miRNAs were overexpressed and 10 miRNAs were underexpressed resulting in a FDR of 1% (Figure 2, Table S1). Among these miRNAs, miR-196b showed the highest increase (67-fold) in the patients with pre-operative seizures when compared with those without pre-operative seizures, so we choose it for further analysis. Furthermore, an independent cohort containing 83 samples was used to validate the relationship of miR-196b and the occurrence of pre-operative seizures. As shown in Figure 3A, patients with pre-operative seizures demonstrated higher expression levels of miR-196b when compared with those without seizures (p<0.001, Student's t-test). To evaluate the possible diagnostic role of miR-196b for occurrence of pre-operative seizures, receiver operating characteristic (ROC) curve analysis was performed (Figure 3B). The area under the curve was 0.965.

### Association of miR-196b expression with seizure control after surgery in patients without pre-operative seizures

**Figure 4:**
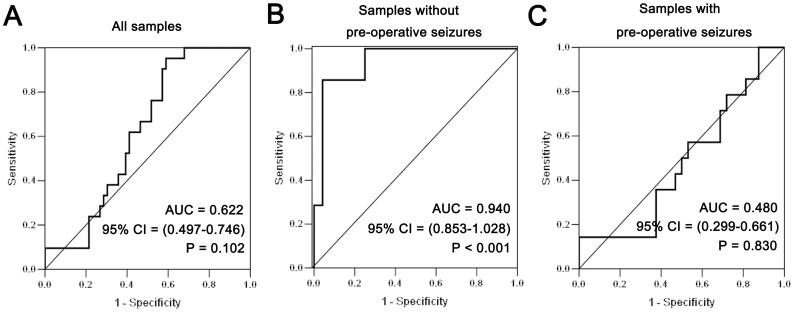
Association of miR-196b expression by qPCR with post-operative seizure control in patients without pre-operative seizures. (A) The predictive power of miR-196b expression in all patients. (B) The predictive power of miR-196b expression in all patients without pre-operative seizures. (C) The predictive power of miR-196b expression in all patients with pre-operative seizures.

In the validation cohort of our study, all patients were subjected to sodium Valproate monotherapy pre- and post-operatively. As shown in Table 2, 32 of 46 patients with follow-up in seizure group and 24 of 32 patients with follow-up in non-seizure group were taken off sodium valproate in 3 months and 2 weeks after surgery respectively. In univariate analysis, extent of tumor resection and expression of miR-196b (p = 0.008 and 0.014, respectively, Chi-square test) were significantly correlated with seizure control in patients without pre-operative seizures. These 2 dichotomous variables were then entered into the multivariate analysis (using logistic regression analysis). Both of them showed significance (p = 0.007 and 0.015, respectively, Table 3). This indicates that higher miR-196b expression levels increased the odds that sodium Valproate treatment in patients without pre-operative seizures would be ineffective. However, in patients with pre-operative seizures, no similar results were observed. The predictive power of miR-196b expression can also be observed in the ROC curve (Figure 4), with an area under the curve of 0.94.

**Table 3:** Multivariate predictors of seizure control at 6 months after surgery in the patients without pre-operation seizures*.

Variable	OR	95%CI	p Value
Gross-total resection	14.680	1.300–165.793	0.007
High miR-196b expression	0.081	0.007–0.936	0.015

* Results of logistic regression analysis.

### Biological Insights of miR-196b

**Figure 5:**
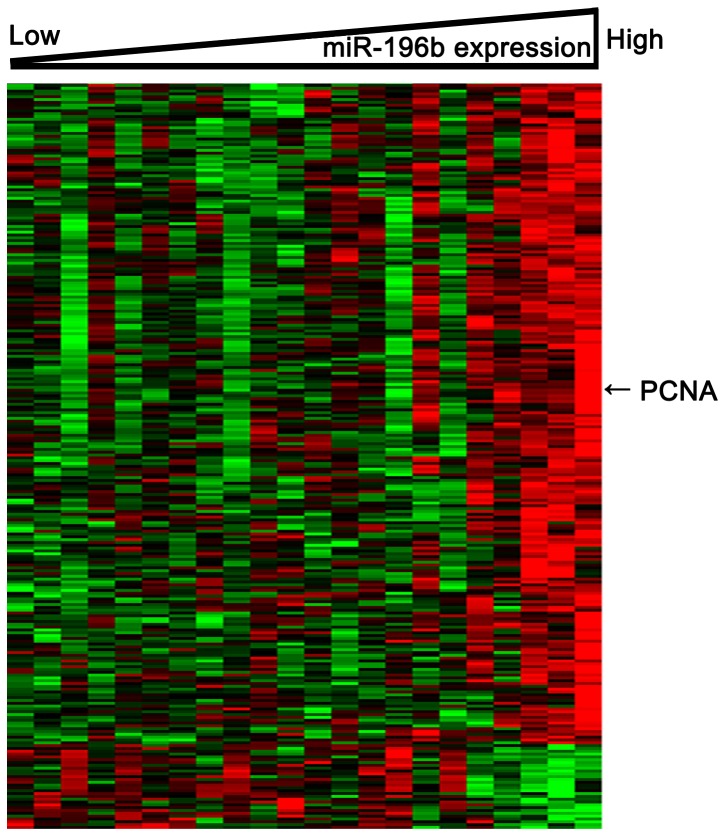
Heat map of the derived gene-expression signature correlated with miR-196b expression. Total 312 negatively correlated probe sets (R<−0.4; p<0.05) and 1111 positively correlated probe sets (R>0.4; p<0.05) with miR-196b were included. Columns represent patients and rows represent probes. Patients are ordered from left to right by increasing miR-196b expression. Probes are ordered by unsupervised clustering using Cluster 3.0. Arrows indicate gene that was discussed in the text (PCNA).

To further understand the potential functional role of miR-196b expression in low-grade gliomas, we performed a GO analysis. Biological processes that relate to transcription and cell cycles were over-represented in the miR-196b-associated gene expression signature (Table 4). To gain insights into the functional contribution of miR-196b expression levels tumor-related epilepsy, we derived a gene expression signature associated with miR-196b expression in 22 patients using whole genome mRNA profiling. We observed that the expression of 1423 probe sets significantly correlated (R>0.4 or <−0.4; p<0.05) with that of miR-196b; 312 probe sets negatively correlated and 1111 probe sets positively correlated (Figure 5, Table S2). Among these genes, we observed a positive correlation of miR-196b expression with the expression of proliferation-associated genes, such as PCNA.

**Table 4:** Biological processes significantly over-represented in the miR-196b expression profile.

GO ID	GO Terms	Count	Fold Enrichment	p Value
0006350	transcription	200	1.890995	5.24E−21
0045449	regulation of transcription	217	1.657318	8.72E−16
0051252	regulation of RNA metabolic process	165	1.807893	6.49E−15
0006355	regulation of transcription, DNA-dependent	161	1.803863	1.96E−14
0051301	cell division	51	3.434272	2.35E−14
0007049	cell cycle	89	2.27832	2.89E−13
0000279	M phase	49	2.958603	2.23E−11
0022403	cell cycle phase	55	2.639057	9.75E−11
0048285	organelle fission	37	3.209613	9.57E−10
0007067	mitosis	36	3.250621	1.18E−09
0000280	nuclear division	36	3.250621	1.18E−09
0022402	cell cycle process	64	2.250184	1.84E−09
0000087	M phase of mitotic cell cycle	36	3.192574	1.94E−09
0000278	mitotic cell cycle	47	2.52338	1.16E−08
0006281	DNA repair	37	2.588033	2.91E−07

## Discussion

Tumor-related epilepsy is among the most common types of symptomatic epilepsy. Many recent studies have demonstrated dysregulated miRNAs in human TLE and post status epilepticus rats [12], [17], [18]. This strongly suggests some miRNA expression patterns may contribute to the epileptogenesis process. Our study provides the first report of quantitative global miRNA expression profiles in low-grade gliomas from clinical tissue samples, and showed an aberrant miRNA expression pattern.

### MiR-196b and tumor malignancy

Currently, microarray profiles of miRNA expression patterns have shown that the highest expression levels of miR-196b were found in human bone marrow and spleen cells [19]. In the central nervous system (CNS), miR-196b has been identified as a candidate oncogene for tumor cells because of the fact that it is expressed at higher levels in CNS tumor-derived cell lines compared with normal brain cells [20]. Increased miR-196 expression levels, relative to their matched normal tissues, have also been reported in a recent study [21], further supporting the relevance of miR-196 to gliomas. In addition, miR-196b may play a role in the malignant progression of gliomas and may be a prognostic predictor in GBMs [13], [22]. These findings suggest that up-regulation of miR-196 could contribute to tumorigenesis and malignant progression, and may have diagnostic and prognostic significance for gliomas. However, there are other reports indicating that miR-196 may play a tumor suppressive role [23], [24].

### MiR-196b and seizures

The mechanisms of action for miR-196 in tumor-related epilepsy are still unknown. These may depend on the biological processes underlying miR-196. In the present study, firstly, miR-196b correlates with seizure occurrence. Now, some studies have demonstrated that patients with epilepsy are more likely to have worse seizure control after surgery than those without pre-operative epilepsy [2]. It is possible that high levels of miR-196b expression, implying a potentially strong proliferation of tumor cells, also suggest a high risk of epileptic susceptibility. Second, miR-196b expression correlates with seizure control. Based on the fact that seizure recurrence is generally associated with tumor progression [2], [25], we hypothesize that proliferation of tumor cells plays a necessary role in seizure occurrence. Thus, if high levels of miR-196b expression cause epilepsy by enhancing the proliferation of tumor cells, it could be reasonably explained that glioma patients with strong miR-196b expression have poor seizure prognosis.

### MiR-196b and cell proliferation

We observed a positive correlation of miR-196b expression with the expression of proliferation-associated genes. This further suggests that the proliferation of tumor cells could possibly play a part in the pathogenesis of tumor-related epilepsy. GO analysis showed that many cell cycle-associated gene sets were over-represented in the miR-196b-associated gene expression signature. The expression of PCNA, a proliferation marker, was also positively correlated with miR-196b expression. These results suggest that miR-196 may obtain its biological role by regulating cell proliferation.

### MiR-196b and Valproate acid

Valproate acid (VPA), a stabilizer of the cell membrane and an inhibitor of GABA receptors controlling epileptic discharge, has the potential for apoptosis, growth arrest, and cell differentiation of tumor cells through inhibition of histone deacetylase [26], [27]. Weller et al. indicated the potential anti-tumor activity of VPA in patients with glioblastoma who require an AED during temozolomide-based chemoradiotherapy [28]. In the present study, miR-196b expression could be considered in some way as a predictor of the efficacy of sodium Valproate in patients without pre-operative epilepsy. The association between miR-196b expression and sodium Valproate response suggests that sodium Valproate mediates the clinical effects of some molecules targeted by miR-196b. The way in which miR-196b regulates the binding of sodium Valproate and leads to seizure reduction is not clear. Additional investigations of functional analysis, particularly on its possible contribution to tumor proliferation, should be conducted to validate the role of miR-196b in tumorigenesis and the malignant progression of glioma. Further understanding of how cell proliferation contributes to the epileptogenesis process will require the development of suitable animal models. If this hypothesis holds, assessment of miR-196b expression in brain tissue (or possibly blood samples in future) of epilepsy patients with glioma might guide the selection of those patients who have a high probability of responding to sodium Valproate.

### Other miRNAs and seizures

Currently, changes in the expression of miR-146a have been implicated in both the development of multiple cancers and in the regulation of inflammation induced via the innate immune response [29], [30]. Aronica et al. indicated that an increased expression of miR-146a was observed in a rat model of TLE as well as in human TLE [12]. Our microarray data also reveals that miR-146a is up-regulated in patients with preoperative seizures. However, further investigation is needed to confirm the role of inflammation in tumor-related seizures.

MiR-15b, another miRNA found in our microarray data, is also reported in several similar studies on GBM [13], [31]. These authors indicated miR-15b was overexpressed in GBM cells compared with normal controls. Moreover, miR-15b was identified in the cerebrospinal fluid as a biomarker for the diagnosis of glioma [32]. Future studies are warranted to characterize its possible mechanism causing seizures.

Additionally, as shown previously, we demonstrated a relationship between extent of resection and seizure prognosis in patients without a history of seizure. It is generally accepted that minimizing the residue volume of tumors with gross total resection could increase the possibility of removing the epileptogenic zone. Thus, patients are more likely to achieve favorable seizure control.

The current study has some limitations. Six or 12 months follow-up is a relatively short period of time for estimating seizure frequency, a longer follow-up would have been preferable when survival information is available. We did not use a control group to evaluate the response to another or newer AEDs such as levetiracetam. Further confirmation on large-scale prospective data would be advantageous.

### Conclusions and perspectives

Our data suggest that high miR-196b expression levels are a novel and valuable marker for the molecular diagnosis and prediction of glioma-related epilepsy. Prospective controlled studies are needed to address this issue. *In vivo* experiments to determine the exact role of miR-196b in tumor proliferation could contribute to explaining epileptogenesis and developing new treatment strategies aimed at improving patients' quality of life.

## Supporting Information

Table S1 **Differently expressed miRNAs between seizure group and non-seizure group.**
(DOC)

Table S2 **Genes significantly correlated with miR-196b expression (R>0.4 or <−0.4; P<0.05).**
(DOC)

## References

[1] Lee JW, Wen PY, Hurwitz S, Black P, kesari S, et al (2010) Morphological characteristics of brain tumors causing seizures. Arch Neurol 67: 336–342. 20212231 10.1001/archneurol.2010.2 PMC2995444

[2] Chang EF, Potts MB, Keles GE, Lamborn KR, Chang SM, et al (2008) Seizure characteristics and control following resection in 332 patients with low-grade gliomas. J Neurosurg 108: 227–235. 18240916 10.3171/JNS/2008/108/2/0227

[3] Danfors T, Ribom D, Berntsson SG, Smits A (2009) Epileptic seizures and survival in early disease of grade 2 gliomas. Eur J Neurol 16: 823–831. 19473357 10.1111/j.1468-1331.2009.02599.x

[4] Schaller B, Rüegg SJ (2003) Brain tumor and seizures: pathophysiology and its implications for treatment revisited. Epilepsia 44: 1223–1232. 12919395 10.1046/j.1528-1157.2003.05203.x

[5] Leighton C, Fisher B, Bauman G, Depiero S, Stitt L, et al (1997) Supratentorial low-grade glioma in adults: an analysis of prognostic factors and timing of radiation. J Clin Oncol 15: 1294–1301. 9193320 10.1200/JCO.1997.15.4.1294

[6] Bartel DP (2004) MicroRNAs: genomics, biogenesis, mechanism and function. Cell 116: 281–297. 14744438 10.1016/s0092-8674(04)00045-5

[7] Pedersen I, David M (2008) MicroRNAs in the immune response. Cytokine 43: 391–394. 18701320 10.1016/j.cyto.2008.07.016 PMC3642994

[8] Pauley KM, Cha S, Chan EK (2009) MicroRNA in autoimmunity and autoimmune diseases. J Autoimmun 32: 189–194. 19303254 10.1016/j.jaut.2009.02.012 PMC2717629

[9] Lukiw WJ (2007) Micro-RNA speciation in fetal, adult and Alzheimer's disease hippocampus. Neuroreport 18: 297–300. 17314675 10.1097/WNR.0b013e3280148e8b

[10] Hébert SS, De Strooper B (2007) Molecular biology miRNAs in neurodegeneration. Science 317: 1179–1180. 17761871 10.1126/science.1148530

[11] Vezzani A, Granata T (2005) Brain inflammation in epilepsy: experimental and clinical evidence. Epilepsia 46: 1724–1743. 16302852 10.1111/j.1528-1167.2005.00298.x

[12] Aronica E, Fluiter K, Lyer A, Zurolo E, Vreijling J, et al (2010) Expression pattern of miR-146a, an inflammation-associated microRNA, in experimental and human temporal lobe epilepsy. Eur J Neurosci 31: 1100–1107. 20214679 10.1111/j.1460-9568.2010.07122.x

[13] Guan Y, Mizoguchi M, Yoshimoto K, Hata N, Shono T, et al (2010) MiRNA-196 is upregulated in glioblastoma but not in anaplastic astrocytoma and has prognostic significance. Clin Cancer Res 16: 4289–4297. 20601442 10.1158/1078-0432.CCR-10-0207

[14] Ma R, Yan W, Zhang G, Lv H, Liu Z, et al (2012) Upregulation of miR-196b Confers a Poor Prognosis in Glioblastoma Patients via Inducing a Proliferative Phenotype. PLoS ONE 7: e38096. 22723849 10.1371/journal.pone.0038096 PMC3378534

[15] Engel J Jr (2001) A proposed diagnostic scheme for people with epileptic seizures and with epilepsy: report of the ILAE Task Force on Classification and Terminology. Epilepsia 42: 796–803. 11422340 10.1046/j.1528-1157.2001.10401.x

[16] Huang da W, Sherman BT, Lempicki RA (2009) Systematic and integrative analysis of large gene lists using DAVID bioinformatics resources. Nat Protoc 4: 44–57. 19131956 10.1038/nprot.2008.211

[17] Song Y, Tian X, Zhang S, Zhang YX, Li X, et al (2011) Temporal Lobe epilepsy induces differential expression of hippocampal miRNAs including let-7e and miR-23a/b. Brain Res 1387: 134–140. 21376023 10.1016/j.brainres.2011.02.073

[18] Hu K, Zhang C, Long L, Long X, Feng L, et al (2011) Expression profile of microRNAs in rat hippocampus following lithium–pilocarpine-induced status epilepticus. Neurosci Lett 488: 252–257. 21094214 10.1016/j.neulet.2010.11.040

[19] Baskerville S, Bartel DP (2005) Microarray profiling of microRNAs reveals frequent coexpression with neighboring miRNAs and host genes. RNA 11: 241–247. 15701730 10.1261/rna.7240905 PMC1370713

[20] Gaur A, Jewell DA, Liang Y, Ridzon D, Moore JH, et al (2007) Characterization of microRNA expression levels and their biological correlates in human cancer cell lines. Cancer Res 67: 2456–2468. 17363563 10.1158/0008-5472.CAN-06-2698

[21] Szafranska AE, Davison TS, John J, Cannon T, Sipos B, et al (2007) MicroRNA expression alterations are linked to tumorigenesis and non-neoplastic processes in pancreatic ductal adenocarcinoma. Oncogene 26: 4442–4452. 17237814 10.1038/sj.onc.1210228

[22] Lakomy R, Sana J, Hankeova S, Fadrus P, Kren L, et al (2011) MiR-195, miR-196b, miR-181c, miR-21 expression levels and O-6-methylguanine-DNA methyltransferase methylation status are associated with clinical outcome in glioblastoma patients. Cancer Sci 102: 2186–2190. 21895872 10.1111/j.1349-7006.2011.02092.x PMC11158343

[23] Braig S, Mueller DW, Rothhammer T, Bosserhoff AK (2010) MicroRNA miR-196a is a central regulator of HOX-B7 and BMP4 expression in malignant melanoma. Cell Mol Life Sci 67: 3535–3548. 20480203 10.1007/s00018-010-0394-7 PMC11115699

[24] Li Y, Zhang M, Chen H, Dong Z, Ganapathy V, et al (2010) Ratio of miR-196s to HOXC8 messenger RNA correlates with breast cancer cell migration and metastasis. Cancer Res 70: 7894–7904. 20736365 10.1158/0008-5472.CAN-10-1675 PMC2955846

[25] Chaichana KL, Parker SL, Olivi A, Quiñones-Hinojosa A (2009) Long-term seizure outcomes in adult patients undergoing primary resection of malignant brain astrocytomas. J Neurosurg 111: 282–292. 19344222 10.3171/2009.2.JNS081132

[26] Eyal S, Yagen B, Sobol E, Altschuler Y, Shmuel M, et al (2004) The activity of antiepileptic drugs as histone deacetylase inhibitors. Epilepsia 45: 737–744. 15230695 10.1111/j.0013-9580.2004.00104.x

[27] Li XN, Shu Q, Su JM, Perlaky, Blaney SM, et al (2005) Valproic acid induces growth arrest, apoptosis, and senescence in medulloblastomas by increasing histone hyperacetylation and regulating expression of p21Cip1, CDK4, and CMYC. Mol Cancer Ther 4: 1912–1922. 16373706 10.1158/1535-7163.MCT-05-0184

[28] Weller M, Gorlia T, Cairncross JG, van den Bent MJ, Mason W, et al (2011) Prolonged survival with valproic acid use in the EORCT/NCIC temozolomide trial for glioblastoma. Neurology 77: 1156–1164. 21880994 10.1212/WNL.0b013e31822f02e1 PMC3265044

[29] Williams AE, Perry MM, Moschos SA, Larner-Svensson HM, Lindsay MA (2008) Role of miRNA-146a in the regulation of the innate immune response and cancer. Biochem Soc Trans 36: 1211–1215. 19021527 10.1042/BST0361211

[30] Nahid MA, Pauley KM, Satoh M, Chan EK (2009) MICRORNA-146A is critical for endotoxin-induced tolerance. Implication on innate immunity. J Biol Chem 284: 34590–34599. 19840932 10.1074/jbc.M109.056317 PMC2787321

[31] Xia H, Qi Y, Ng SS, et al (2009) MicroRNA-15b regulates cell cycle progression by targeting cyclins in glioma cells. Biochem Biophys Res Commun 380: 205–210. 19135980 10.1016/j.bbrc.2008.12.169

[32] Baraniskin A, Kuhnhenn J, Schlegel U, Maghnouj A, Zöllner H, et al (2012) Identification of microRNAs in the cerebrospinal fluid as biomarker for the diagnosis of glioma. Neuro Oncol 14: 29–23. 21937590 10.1093/neuonc/nor169 PMC3245991

