# Supplementary material for: Significance of miR-196b in Tumor-Related Epilepsy of Patients with Gliomas
Source: PLoS One. 2012 Sep 25;7(9):e46218. doi: 10.1371/journal.pone.0046218 (PMC3457999; doi:10.1371/journal.pone.0046218)
Supplement: Table S1 — Differently expressed miRNAs between seizure group and non-seizure group. (DOC) [file pone.0046218.s001.doc]

**Table S1:** Differently expressed miRNAs between seizure group and non-seizure group.

| Gene ID | Gene Name | Fold Change | Q Value |
| --- | --- | --- | --- |
| ILMN_3168308 | hsa-miR-196b | 67.29075053 | < 0.01 |
| ILMN_3168555 | hsa-miR-181c* | 31.60170217 | < 0.01 |
| ILMN_3166998 | hsa-miR-340* | 7.51887866 | < 0.01 |
| ILMN_3168672 | hsa-miR-16-2* | 6.827145026 | < 0.01 |
| ILMN_3168621 | hsa-miR-22* | 5.733390417 | < 0.01 |
| ILMN_3168783 | hsa-miR-500 | 5.602175669 | < 0.01 |
| ILMN_3168755 | hsa-miR-29b-1* | 4.726560644 | < 0.01 |
| ILMN_3167305 | hsa-miR-652 | 4.681148699 | < 0.01 |
| ILMN_3167441 | hsa-miR-192 | 4.442425492 | < 0.01 |
| ILMN_3167122 | hsa-miR-194 | 3.568522982 | < 0.01 |
| ILMN_3167437 | hsa-miR-497 | 3.499753049 | < 0.01 |
| ILMN_3168749 | hsa-miR-455-3p | 3.138264249 | < 0.01 |
| ILMN_3168344 | hsa-miR-363 | 2.937989723 | < 0.01 |
| ILMN_3168588 | hsa-miR-130b* | 2.771197415 | < 0.01 |
| ILMN_3167491 | hsa-miR-128b:9.1 | 2.587852732 | < 0.01 |
| ILMN_3168319 | hsa-miR-454 | 2.443482349 | < 0.01 |
| ILMN_3168102 | hsa-miR-502-3p/500* | 2.426243016 | < 0.01 |
| ILMN_3168885 | hsa-miR-335* | 2.346256188 | < 0.01 |
| ILMN_3168085 | hsa-miR-128a:9.1 | 2.343171475 | < 0.01 |
| ILMN_3167060 | hsa-miR-15b | 2.341022 | < 0.01 |
| ILMN_3168425 | hsa-miR-598 | 2.268752828 | < 0.01 |
| ILMN_3168483 | hsa-miR-146a | 2.265967867 | < 0.01 |
| ILMN_3168273 | hsa-miR-503 | 2.200295325 | < 0.01 |
| ILMN_3168262 | hsa-miR-99b | 2.1860532 | < 0.01 |
| ILMN_3167455 | hsa-miR-30a | 2.166070625 | < 0.01 |
| ILMN_3167006 | hsa-miR-378 | 2.153689958 | < 0.01 |
| ILMN_3168213 | hsa-miR-99a | 2.118091375 | < 0.01 |
| ILMN_3168604 | hsa-miR-1201 | 2.107524509 | < 0.01 |
| ILMN_3167699 | hsa-let-7b* | 2.010435974 | < 0.01 |
| ILMN_3167158 | hsa-miR-30a* | 2.003541969 | < 0.01 |
| ILMN_3167132 | hsa-miR-517a/b | 0.064774551 | < 0.01 |
| ILMN_3167241 | hsa-miR-518b | 0.074621773 | < 0.01 |
| ILMN_3167141 | hsa-miR-622 | 0.236295092 | < 0.01 |
| ILMN_3168801 | hsa-miR-654-3p | 0.252033912 | < 0.01 |
| ILMN_3168840 | hsa-miR-18b* | 0.28550049 | < 0.01 |
| ILMN_3168322 | hsa-miR-302b* | 0.29968893 | < 0.01 |
| ILMN_3167386 | hsa-miR-302d | 0.318513433 | < 0.01 |
| ILMN_3168451 | hsa-miR-346 | 0.38617162 | < 0.01 |
| ILMN_3168485 | hsa-miR-504 | 0.435691131 | < 0.01 |
| ILMN_3168564 | hsa-miR-940 | 0.488212766 | < 0.01 |
