# Supplementary material for: Significance of miR-196b in Tumor-Related Epilepsy of Patients with Gliomas
Source: PLoS One. 2012 Sep 25;7(9):e46218. doi: 10.1371/journal.pone.0046218 (PMC3457999; doi:10.1371/journal.pone.0046218)
Supplement: Table S2 — Genes significantly correlated with miR-196b expression (R>0.4 or <−0.4; P<0.05). (DOC) [file pone.0046218.s002.doc]

**Table S2:** Genes significantly correlated with miR-196b expression (R > 0.4 or < -0.4; P < 0.05)

| Gene Symbol | Probe Name | R Value | P Value |
| --- | --- | --- | --- |
| HRASLS | A_23_P57658 | -0.729064379 | 0.000118567 |
| IL18R1 | A_24_P208567 | -0.694268821 | 0.000337628 |
| TMEM169 | A_24_P929388 | -0.657208998 | 0.000889693 |
| CPNE5 | A_23_P360804 | -0.646786749 | 0.001142029 |
| AMZ1 | A_24_P383649 | -0.643774751 | 0.001225408 |
| PDE8B | A_24_P197537 | -0.625446727 | 0.001852694 |
| LOC100130107 | A_32_P1516 | -0.609272066 | 0.002613826 |
| MCTP1 | A_23_P133293 | -0.600998001 | 0.003095417 |
| HSPC159 | A_23_P210330 | -0.571126723 | 0.005498841 |
| MCTP1 | A_24_P212481 | -0.570433078 | 0.005569201 |
| CSPG4 | A_23_P21976 | -0.565527607 | 0.006088459 |
| LRFN5 | A_23_P163195 | -0.562252546 | 0.006457012 |
| SP5 | A_23_P135381 | -0.554637469 | 0.007385895 |
| AFAP1L2 | A_23_P406341 | -0.55208236 | 0.007721266 |
| ACSL4 | A_23_P217564 | -0.54976923 | 0.008035625 |
| VPS26B | A_23_P52903 | -0.549357321 | 0.008092696 |
| TOMM70A | A_23_P144264 | -0.549222017 | 0.008111516 |
| PPP1R14C | A_23_P45011 | -0.548245141 | 0.008248465 |
| PCTK2 | A_23_P33376 | -0.547813795 | 0.008309539 |
| KCNJ11 | A_23_P1973 | -0.545889413 | 0.008586572 |
| KIF5C | A_32_P154473 | -0.544040892 | 0.008859805 |
| TYRO3 | A_23_P54517 | -0.544025302 | 0.008862139 |
| CXADR | A_32_P29632 | -0.544022644 | 0.008862537 |
| MEIS3P1 | A_24_P648880 | -0.543728727 | 0.008906645 |
| TMEM169 | A_23_P68338 | -0.542140642 | 0.009148102 |
| GRASP | A_23_P105442 | -0.541685992 | 0.009218212 |
| IGSF21 | A_32_P78101 | -0.54153183 | 0.009242084 |
| GRB10 | A_24_P235266 | -0.540475344 | 0.009407057 |
| ESYT2 | A_24_P132470 | -0.539654834 | 0.009536844 |
| SCG5 | A_23_P62081 | -0.538274121 | 0.009758564 |
| NGFRAP1 | A_23_P45524 | -0.536461304 | 0.010056083 |
| GPR17 | A_23_P16963 | -0.53589899 | 0.010149867 |
| DMD | A_24_P34186 | -0.534466516 | 0.010392027 |
| LOR | A_23_P34452 | -0.532867072 | 0.010667989 |
| MORF4L2 | A_24_P333445 | -0.530947988 | 0.011006997 |
| TCEAL8 | A_23_P11331 | -0.530122412 | 0.011155523 |
| C1QL2 | A_23_P50928 | -0.529977526 | 0.011181758 |
| FAM131B | A_24_P252310 | -0.529082137 | 0.011345008 |
| ZNF488 | A_23_P23966 | -0.529009133 | 0.011358403 |
| AGAP2 | A_24_P244495 | -0.522533887 | 0.012599129 |
| C5orf25 | A_24_P392060 | -0.52201669 | 0.012702816 |
| DACH2 | A_32_P464135 | -0.521209139 | 0.012866103 |
| FAM19A1 | A_32_P117693 | -0.519438616 | 0.013230098 |
| ADRA1B | A_23_P33326 | -0.519053246 | 0.013310426 |
| ANO2 | A_32_P141238 | -0.517865968 | 0.013560407 |
| MYT1L | A_32_P91663 | -0.517846299 | 0.01356458 |
| SNHG11 | A_23_P325119 | -0.516079643 | 0.013943708 |
| DLK1 | A_24_P236251 | -0.514934971 | 0.014193927 |
| SHANK3 | A_24_P194881 | -0.513300225 | 0.014557592 |
| CDIPT | A_23_P501339 | -0.513139305 | 0.014593796 |
| STMN2 | A_23_P146274 | -0.511457554 | 0.014976551 |
| SLC7A14 | A_32_P88262 | -0.510962987 | 0.015090649 |
| NIPSNAP1 | A_24_P237278 | -0.510840964 | 0.015118908 |
| NIPSNAP1 | A_23_P120860 | -0.509663129 | 0.015393896 |
| LZTS1 | A_23_P20443 | -0.508324548 | 0.015711325 |
| USP6 | A_24_P170454 | -0.506297306 | 0.016202148 |
| SFRS2B | A_32_P17635 | -0.506089433 | 0.016253171 |
| FAM134A | A_23_P108522 | -0.505696035 | 0.016350087 |
| HSPC159 | A_23_P430818 | -0.505683179 | 0.016353263 |
| DOK4 | A_23_P77381 | -0.504395095 | 0.016673922 |
| CXADR | A_23_P57268 | -0.501503968 | 0.017412193 |
| GFOD1 | A_23_P122662 | -0.500641743 | 0.01763741 |
| CNTNAP5 | A_23_P56787 | -0.499968851 | 0.017814802 |
| C1orf173 | A_24_P634867 | -0.498946858 | 0.01808698 |
| TFAP2E | A_23_P322116 | -0.49856869 | 0.01818854 |
| FLJ43390 | A_32_P227216 | -0.498411136 | 0.018230989 |
| 4-Mar | A_23_P333228 | -0.495960344 | 0.018901638 |
| YPEL4 | A_23_P300220 | -0.495584932 | 0.019006102 |
| STOML1 | A_23_P54376 | -0.495270799 | 0.019093871 |
| DOK4 | A_24_P48069 | -0.493622315 | 0.019559827 |
| NEURL1B | A_32_P198731 | -0.492184752 | 0.019973598 |
| KIFC3 | A_23_P54576 | -0.491815875 | 0.020080899 |
| LPCAT1 | A_23_P213471 | -0.490844794 | 0.020365589 |
| LOC285033 | A_24_P687131 | -0.490744547 | 0.020395162 |
| KCNMB2 | A_23_P170072 | -0.490711297 | 0.020404979 |
| SLC7A14 | A_23_P253451 | -0.490502206 | 0.020466796 |
| PRKCD | A_23_P144054 | -0.490299842 | 0.020526768 |
| ARID5B | A_32_P18440 | -0.489860782 | 0.020657372 |
| FLJ23867 | A_23_P380266 | -0.489740876 | 0.020693155 |
| NGEF | A_23_P102364 | -0.489656188 | 0.020718459 |
| C9orf24 | A_23_P217009 | -0.488842833 | 0.020962743 |
| EIF3C | A_23_P77568 | -0.488354349 | 0.021110564 |
| SCGB3A2 | A_23_P145006 | -0.487999056 | 0.021218604 |
| KIAA1409 | A_23_P432272 | -0.487994455 | 0.021220006 |
| WFDC1 | A_23_P106617 | -0.48743561 | 0.021390852 |
| TCEAL5 | A_32_P18470 | -0.486839911 | 0.021574177 |
| SCG2 | A_24_P88696 | -0.485062364 | 0.022128721 |
| LOC100288144 | A_32_P109755 | -0.484493074 | 0.022308718 |
| NEURL | A_23_P322562 | -0.483954125 | 0.022480203 |
| CAMKK1 | A_23_P431933 | -0.483382844 | 0.022663126 |
| RNF112 | A_23_P107116 | -0.48317202 | 0.022730931 |
| B3GAT1 | A_23_P1833 | -0.481654265 | 0.023223886 |
| SLC2A3 | A_23_P139669 | -0.481390193 | 0.023310521 |
| RIMS2 | A_24_P222000 | -0.481182746 | 0.023378761 |
| PCDH20 | A_23_P204885 | -0.480158812 | 0.023717928 |
| DIO2 | A_23_P48740 | -0.479224674 | 0.024030773 |
| SHC3 | A_23_P61406 | -0.478994563 | 0.024108341 |
| TEKT4 | A_32_P231179 | -0.478928696 | 0.024130581 |
| MGC4859 | A_24_P709536 | -0.47859698 | 0.024242835 |
| PHYHIP | A_23_P433229 | -0.478577431 | 0.024249463 |
| UNC80 | A_24_P817863 | -0.478517075 | 0.024269937 |
| SP5 | A_32_P183718 | -0.477316593 | 0.024680041 |
| TRNT1 | A_23_P372331 | -0.476784796 | 0.024863471 |
| FLJ43390 | A_24_P840042 | -0.476045421 | 0.025120306 |
| OPCML | A_23_P72663 | -0.475630599 | 0.025265326 |
| BAI2 | A_23_P149019 | -0.475623149 | 0.025267937 |
| EGR3 | A_23_P216225 | -0.474884812 | 0.02552773 |
| RALYL | A_23_P388220 | -0.474591634 | 0.025631477 |
| ASPHD1 | A_23_P170667 | -0.47383043 | 0.025902416 |
| SLC38A1 | A_24_P261734 | -0.473609769 | 0.025981382 |
| PDE5A | A_24_P911678 | -0.473341367 | 0.026077691 |
| C18orf45 | A_23_P89824 | -0.472674836 | 0.026318089 |
| STXBP5L | A_23_P418785 | -0.472656987 | 0.026324551 |
| LPAR3 | A_23_P436048 | -0.472447633 | 0.026400437 |
| FAM84A | A_23_P306211 | -0.471416962 | 0.026776575 |
| ANKRD43 | A_23_P304110 | -0.4713564 | 0.026798808 |
| CAND2 | A_23_P250102 | -0.471315202 | 0.026813942 |
| CNTNAP5 | A_23_P373109 | -0.470794267 | 0.027005883 |
| PCOLCE2 | A_23_P57709 | -0.470222508 | 0.027217808 |
| CHRM3 | A_24_P817236 | -0.470184024 | 0.027232119 |
| RP1-21O18.1 | A_24_P331830 | -0.468838796 | 0.027736163 |
| CYP2C19 | A_23_P158484 | -0.468449513 | 0.027883399 |
| DLG3 | A_24_P945283 | -0.468414659 | 0.027896611 |
| LOR | A_24_P171274 | -0.468198841 | 0.027978537 |
| STOML1 | A_23_P48910 | -0.467529408 | 0.028233873 |
| FAIM2 | A_23_P139891 | -0.46670348 | 0.028551446 |
| LOC100128239 | A_32_P115438 | -0.466540658 | 0.028614385 |
| HYAL3 | A_23_P361405 | -0.466163493 | 0.028760602 |
| RAB9B | A_24_P126139 | -0.466099662 | 0.028785406 |
| ARX | A_23_P306223 | -0.465974976 | 0.028833908 |
| RASGEF1C | A_24_P383762 | -0.465791793 | 0.02890528 |
| ALPPL2 | A_24_P131580 | -0.465652271 | 0.028959736 |
| STAC2 | A_32_P152195 | -0.465267143 | 0.029110474 |
| ZNF365 | A_24_P226970 | -0.464794419 | 0.029296347 |
| SYT14 | A_24_P402415 | -0.464514847 | 0.029406716 |
| CYCS | A_24_P573978 | -0.463890908 | 0.029654222 |
| XAGE2B | A_23_P34031 | -0.463667308 | 0.029743321 |
| SFRS2B | A_23_P116016 | -0.463270871 | 0.029901813 |
| CACNG8 | A_23_P420218 | -0.462581226 | 0.030179122 |
| BAI1 | A_23_P125056 | -0.462496901 | 0.030213168 |
| ZNF365 | A_23_P86610 | -0.461764791 | 0.030510042 |
| ATP1A4 | A_24_P307626 | -0.461490624 | 0.03062181 |
| SDR16C5 | A_24_P317708 | -0.46136878 | 0.030671586 |
| ATP1A1 | A_23_P1072 | -0.460911725 | 0.030858872 |
| CHRD | A_24_P117725 | -0.460638046 | 0.030971449 |
| NCAM2 | A_32_P199429 | -0.460107886 | 0.031190454 |
| OLFM1 | A_23_P219161 | -0.459397085 | 0.031486 |
| RSPO2 | A_23_P258294 | -0.459378517 | 0.03149375 |
| RP1-21O18.1 | A_24_P246573 | -0.459243615 | 0.031550102 |
| MED13L | A_23_P331028 | -0.458862393 | 0.031709777 |
| TTC7B | A_24_P127292 | -0.458419638 | 0.031896027 |
| ACVR1C | A_32_P190944 | -0.457904308 | 0.032113894 |
| ATCAY | A_23_P433063 | -0.457143623 | 0.032437638 |
| P2RX2 | A_23_P124003 | -0.456699226 | 0.03262796 |
| DNAH8 | A_24_P926631 | -0.456198847 | 0.032843311 |
| ANK3 | A_23_P301530 | -0.456191371 | 0.032846537 |
| C3orf10 | A_23_P420361 | -0.456098978 | 0.032886427 |
| RABGEF1 | A_24_P337746 | -0.455988385 | 0.032934224 |
| CPLX2 | A_24_P391868 | -0.455728172 | 0.033046902 |
| NRXN3 | A_23_P251841 | -0.455670757 | 0.033071804 |
| ACSBG1 | A_23_P54488 | -0.455443217 | 0.033170642 |
| WNT7B | A_23_P320054 | -0.455188054 | 0.033281755 |
| SLC4A3 | A_23_P39647 | -0.453398215 | 0.034069433 |
| PTPRN | A_23_P90722 | -0.452655444 | 0.034400596 |
| SYNPO | A_23_P213798 | -0.452512622 | 0.034464563 |
| CBX6 | A_24_P254437 | -0.452473461 | 0.034482118 |
| C22orf9 | A_32_P88479 | -0.452159498 | 0.03462312 |
| PGRMC1 | A_23_P114275 | -0.452118294 | 0.034641659 |
| PDE4A | A_24_P322474 | -0.451158267 | 0.035075812 |
| RIMS2 | A_23_P147786 | -0.451065734 | 0.035117883 |
| NRXN3 | A_23_P254934 | -0.451033096 | 0.035132732 |
| ELL2 | A_23_P58506 | -0.450914563 | 0.035186701 |
| MLXIPL | A_24_P200831 | -0.450861754 | 0.035210766 |
| GFOD1 | A_32_P15035 | -0.450749982 | 0.035261744 |
| MXRA7 | A_32_P161681 | -0.450554945 | 0.035350836 |
| TCEAL7 | A_24_P49106 | -0.45054498 | 0.035355393 |
| CAPN13 | A_23_P101972 | -0.450339453 | 0.035449478 |
| PTGIS | A_24_P48723 | -0.449499887 | 0.035835858 |
| CHL1 | A_24_P63178 | -0.449100284 | 0.03602092 |
| GALNT9 | A_23_P252462 | -0.448945443 | 0.036092831 |
| FAM13B | A_23_P136460 | -0.448612256 | 0.036247952 |
| NPTX2 | A_23_P82651 | -0.448605113 | 0.036251283 |
| RIMS2 | A_24_P332816 | -0.448264134 | 0.036410584 |
| UFM1 | A_24_P313109 | -0.448119761 | 0.036478199 |
| NEK1 | A_24_P50882 | -0.448085897 | 0.036494073 |
| TMEM130 | A_23_P349966 | -0.447999315 | 0.036534683 |
| OPCML | A_32_P160563 | -0.447462175 | 0.036787418 |
| CXADRP2 | A_32_P125135 | -0.447160222 | 0.036930094 |
| LIMD2 | A_24_P159635 | -0.447082333 | 0.036966968 |
| C2orf55 | A_24_P272313 | -0.446948347 | 0.037030467 |
| MGAT3 | A_24_P245838 | -0.44682821 | 0.037087474 |
| SLC38A1 | A_23_P363399 | -0.446582405 | 0.03720433 |
| GJB5 | A_23_P35293 | -0.446265156 | 0.037355577 |
| ANKH | A_24_P303145 | -0.446264959 | 0.03735567 |
| ACCN1 | A_32_P159612 | -0.445708676 | 0.03762204 |
| KSR2 | A_32_P216635 | -0.444886596 | 0.038018409 |
| KIAA0284 | A_23_P48550 | -0.444847858 | 0.038037167 |
| C12orf44 | A_23_P116840 | -0.444776553 | 0.038071714 |
| RAP1GAP | A_24_P36890 | -0.444018152 | 0.038440681 |
| SGK493 | A_32_P112493 | -0.443715271 | 0.038588815 |
| CASP2 | A_24_P269398 | -0.443654709 | 0.038618488 |
| SNCA | A_23_P29939 | -0.443337011 | 0.038774443 |
| ARID5B | A_24_P170667 | -0.443208181 | 0.038837825 |
| ARMC8 | A_24_P187954 | -0.442771482 | 0.039053276 |
| GPRASP1 | A_23_P96590 | -0.442751421 | 0.039063196 |
| MORF4L2 | A_23_P114405 | -0.442728268 | 0.039074647 |
| TOMM22 | A_23_P80336 | -0.442604818 | 0.039135748 |
| RBM8A | A_23_P305335 | -0.442571517 | 0.039152243 |
| HPCAL1 | A_23_P5831 | -0.442190458 | 0.039341381 |
| IDS | A_23_P217475 | -0.442159301 | 0.039356877 |
| IDH3A | A_24_P167806 | -0.441829345 | 0.039521278 |
| LGI1 | A_23_P127267 | -0.441772666 | 0.039549573 |
| KIAA1644 | A_32_P37867 | -0.4416276 | 0.039622063 |
| PPARGC1B | A_24_P560519 | -0.441291789 | 0.039790267 |
| ARL6IP4 | A_23_P47848 | -0.441259886 | 0.039806276 |
| PICK1 | A_23_P91727 | -0.440894721 | 0.039989874 |
| C3orf26 | A_23_P132874 | -0.44073411 | 0.040070836 |
| C20orf46 | A_23_P120504 | -0.440717311 | 0.040079311 |
| RIT2 | A_23_P170050 | -0.440319601 | 0.040280376 |
| KCNK1 | A_23_P126075 | -0.439951596 | 0.040467126 |
| CDH9 | A_23_P92999 | -0.439732443 | 0.040578659 |
| LOC100132288 | A_24_P306443 | -0.439481934 | 0.040706444 |
| EPHB6 | A_23_P145935 | -0.438801396 | 0.041055177 |
| OR7A17 | A_23_P28023 | -0.438645905 | 0.041135182 |
| UNC13C | A_23_P88576 | -0.438522308 | 0.041198864 |
| C1orf212 | A_23_P314666 | -0.438432245 | 0.041245316 |
| NEFM | A_24_P264832 | -0.438281085 | 0.041323373 |
| AMACR | A_24_P106297 | -0.437927819 | 0.041506244 |
| YPEL4 | A_24_P21770 | -0.437884327 | 0.041528802 |
| TCOF1 | A_24_P243396 | -0.437786718 | 0.041579462 |
| 1-Mar | A_24_P354412 | -0.437765255 | 0.041590609 |
| PPFIBP1 | A_23_P373724 | -0.437566737 | 0.041693814 |
| FAM149A | A_32_P130915 | -0.437546151 | 0.041704527 |
| UNC13A | A_24_P472007 | -0.437375802 | 0.041793265 |
| FAIM2 | A_24_P275428 | -0.43736993 | 0.041796326 |
| BEGAIN | A_23_P72127 | -0.437325767 | 0.041819357 |
| LOC344967 | A_24_P698141 | -0.437249534 | 0.041859134 |
| NTNG1 | A_23_P201547 | -0.437024579 | 0.041976687 |
| KCNK10 | A_23_P65629 | -0.436155937 | 0.042433024 |
| FLJ36848 | A_24_P247039 | -0.436074104 | 0.042476213 |
| AES | A_23_P165061 | -0.436065116 | 0.042480959 |
| EXTL1 | A_23_P62999 | -0.435980805 | 0.042525495 |
| ZNF385D | A_23_P113748 | -0.435907458 | 0.04256427 |
| PPP2R2C | A_24_P3415 | -0.435104503 | 0.042990555 |
| NELL2 | A_23_P10025 | -0.435051846 | 0.043018626 |
| WDR88 | A_32_P346172 | -0.43481598 | 0.04314454 |
| SCYL1 | A_24_P30314 | -0.434771519 | 0.043168307 |
| TSHZ3 | A_23_P361014 | -0.434291519 | 0.043425546 |
| KCNMB2 | A_23_P158675 | -0.434109726 | 0.043523283 |
| CAMKV | A_23_P29680 | -0.434108914 | 0.04352372 |
| TMEM35 | A_23_P45324 | -0.433890672 | 0.043641279 |
| TNNT1 | A_23_P56050 | -0.433322427 | 0.043948534 |
| C14orf132 | A_24_P920447 | -0.432993367 | 0.044127227 |
| SNAP25 | A_23_P210756 | -0.432845749 | 0.044207574 |
| P4HTM | A_23_P113317 | -0.432811918 | 0.044226003 |
| TCEAL3 | A_23_P434442 | -0.432504802 | 0.044393582 |
| SEMA3E | A_23_P59582 | -0.431738106 | 0.044814085 |
| ACOT7 | A_24_P205589 | -0.431706848 | 0.044831295 |
| GPR26 | A_23_P415541 | -0.431369348 | 0.045017436 |
| MKL2 | A_23_P54556 | -0.430804674 | 0.045330212 |
| LOC401097 | A_23_P305245 | -0.430772961 | 0.045347828 |
| MEG3 | A_24_P737660 | -0.430760839 | 0.045354562 |
| ENTPD3 | A_23_P212469 | -0.430541222 | 0.045476716 |
| MAP3K10 | A_24_P284523 | -0.430492564 | 0.045503815 |
| KIAA1045 | A_23_P319232 | -0.43038961 | 0.045561193 |
| C7orf40 | A_24_P506977 | -0.430224854 | 0.045653132 |
| PGAM5 | A_23_P319719 | -0.429756354 | 0.045915358 |
| GRIN2A | A_32_P169114 | -0.429566984 | 0.046021682 |
| SEZ6L2 | A_24_P116587 | -0.428913305 | 0.046390168 |
| C1orf14 | A_23_P136571 | -0.428823166 | 0.04644116 |
| PRKCZ | A_23_P51187 | -0.42880276 | 0.04645271 |
| MED10 | A_23_P124022 | -0.428751887 | 0.046481513 |
| MEG3 | A_24_P272993 | -0.428457288 | 0.046648583 |
| TCEAL6 | A_32_P192545 | -0.428320256 | 0.046726454 |
| MLXIPL | A_23_P145786 | -0.428250956 | 0.046765873 |
| CABYR | A_23_P314712 | -0.428135575 | 0.046831562 |
| SAMD14 | A_24_P185186 | -0.428069526 | 0.046869197 |
| ARMCX3 | A_23_P217611 | -0.427958043 | 0.046932774 |
| ACSL3 | A_24_P248606 | -0.427760121 | 0.04704581 |
| LRRC2 | A_23_P334798 | -0.427429503 | 0.047235102 |
| CD99L2 | A_23_P253052 | -0.42737817 | 0.047264545 |
| DIO3 | A_23_P105923 | -0.427335961 | 0.047288766 |
| VSNL1 | A_23_P209978 | -0.427186426 | 0.04737465 |
| PDE4DIP | A_23_P149153 | -0.427088933 | 0.047430709 |
| RBPJL | A_23_P346884 | -0.42698382 | 0.047491207 |
| GK | A_24_P100382 | -0.426449558 | 0.04779963 |
| NCKAP5L | A_24_P177964 | -0.4262545 | 0.04791262 |
| ZNF702P | A_24_P344516 | -0.426192546 | 0.047948552 |
| ATP2B1 | A_23_P128319 | -0.426172955 | 0.047959918 |
| THY1 | A_23_P36364 | -0.426122059 | 0.047989457 |
| KCNH2 | A_23_P377882 | -0.425946524 | 0.048091442 |
| DMD | A_24_P185854 | -0.425909338 | 0.048113069 |
| LOC100289297 | A_32_P208915 | -0.425831876 | 0.048158143 |
| SEZ6L2 | A_23_P10194 | -0.425482043 | 0.048362113 |
| CEND1 | A_23_P170030 | -0.425169585 | 0.048544857 |
| FFAR3 | A_23_P56239 | -0.425099531 | 0.048585902 |
| CXADRP3 | A_24_P161725 | -0.425028693 | 0.048627434 |
| AZI2 | A_24_P105913 | -0.424941523 | 0.048678578 |
| PADI4 | A_23_P138262 | -0.424930669 | 0.048684949 |
| C12orf68 | A_23_P344194 | -0.424843153 | 0.048736344 |
| LOC283089 | A_24_P484791 | -0.424806775 | 0.04875772 |
| ZNF385D | A_24_P234554 | -0.424758569 | 0.048786057 |
| CDRT4 | A_23_P427083 | -0.424176085 | 0.049129469 |
| TLE2 | A_23_P153676 | -0.424068781 | 0.049192935 |
| MAFK | A_23_P373598 | -0.424029716 | 0.049216056 |
| C1orf173 | A_32_P84237 | -0.423725165 | 0.049396597 |
| MPND | A_24_P44931 | -0.423703201 | 0.049409637 |
| SMAP2 | A_23_P406330 | -0.42356866 | 0.049489574 |
| ASB6 | A_23_P158059 | -0.423450993 | 0.049559567 |
| EFHD2 | A_23_P23443 | -0.422998881 | 0.049829214 |
| KCNMA1 | A_32_P192692 | -0.422937974 | 0.049865626 |
| CBX6 | A_24_P370372 | -0.422821813 | 0.049935129 |
| MLF1 | A_23_P419947 | 0.422753402 | 0.049976095 |
| SFXN1 | A_24_P394420 | 0.422763865 | 0.049969829 |
| CSNK2B | A_24_P804263 | 0.42280423 | 0.049945656 |
| SH3BGRL | A_23_P148297 | 0.422823711 | 0.049933992 |
| SEMA6A | A_23_P312840 | 0.422989119 | 0.049835048 |
| CUL4B | A_23_P422178 | 0.423044589 | 0.049801902 |
| SFRS12IP1 | A_32_P198330 | 0.423101791 | 0.049767737 |
| RFX6 | A_23_P327462 | 0.42326184 | 0.049672243 |
| RPP21 | A_23_P214594 | 0.423310506 | 0.049643234 |
| ZNF160 | A_24_P16950 | 0.423435189 | 0.049568974 |
| RDBP | A_23_P122545 | 0.42350769 | 0.049525832 |
| KGFLP1 | A_32_P11372 | 0.42362967 | 0.049453313 |
| BUB1B | A_23_P163481 | 0.423677834 | 0.049424702 |
| MGA | A_23_P37654 | 0.423686601 | 0.049419495 |
| STK4 | A_24_P94054 | 0.423827941 | 0.049335613 |
| TRIP13 | A_24_P277576 | 0.42390843 | 0.049287894 |
| TIMM8B | A_23_P98382 | 0.423918319 | 0.049282034 |
| CCDC81 | A_23_P128008 | 0.423940972 | 0.049268612 |
| AGBL3 | A_24_P374572 | 0.423966771 | 0.049253328 |
| ATRN | A_23_P79931 | 0.42414632 | 0.049147067 |
| CHML | A_24_P712350 | 0.424229327 | 0.049098002 |
| FOXP2 | A_24_P921683 | 0.424327339 | 0.049040116 |
| ASPH | A_24_P18105 | 0.424355916 | 0.049023248 |
| SLC25A33 | A_23_P36226 | 0.424372259 | 0.049013603 |
| PRKAB2 | A_23_P350704 | 0.424430473 | 0.048979262 |
| EFEMP1 | A_23_P501007 | 0.424467888 | 0.048957199 |
| SAC3D1 | A_23_P348298 | 0.424493222 | 0.048942265 |
| LOC100190939 | A_23_P326987 | 0.424499818 | 0.048938377 |
| POLI | A_23_P306890 | 0.424528455 | 0.048921501 |
| ELMO2 | A_24_P322635 | 0.424535848 | 0.048917145 |
| UBE2V1 | A_24_P5935 | 0.424626276 | 0.048863888 |
| FANCI | A_32_P95729 | 0.424644448 | 0.048853192 |
| EFHA1 | A_23_P76598 | 0.424768398 | 0.048780278 |
| LOC100133161 | A_24_P818010 | 0.424802443 | 0.048760266 |
| RBM22 | A_23_P92895 | 0.42484289 | 0.048736499 |
| SRP9 | A_24_P66528 | 0.424893108 | 0.048707002 |
| PTCRA | A_23_P30755 | 0.424910547 | 0.048696763 |
| WDR19 | A_24_P394361 | 0.425105117 | 0.048582628 |
| CDKL3 | A_23_P110643 | 0.42513229 | 0.048566705 |
| PEX12 | A_24_P416411 | 0.425140029 | 0.048562171 |
| POLR3H | A_24_P322847 | 0.425141562 | 0.048561272 |
| BTBD12 | A_23_P371129 | 0.425254149 | 0.048495346 |
| ZNF41 | A_23_P45234 | 0.425279659 | 0.048480418 |
| FCHO2 | A_23_P349083 | 0.425308917 | 0.048463301 |
| ZFP37 | A_23_P254025 | 0.425385102 | 0.048418753 |
| TOP3A | A_23_P15829 | 0.425417581 | 0.04839977 |
| PLA2G4C | A_23_P50508 | 0.425447905 | 0.048382053 |
| MCM3 | A_23_P7873 | 0.42544887 | 0.048381489 |
| KDM1B | A_23_P338401 | 0.425548757 | 0.048323164 |
| H3F3A | A_23_P23141 | 0.425686159 | 0.048243022 |
| ZNF738 | A_32_P81806 | 0.42571587 | 0.048225706 |
| C1orf110 | A_24_P6370 | 0.425802378 | 0.048175316 |
| UBAP2 | A_23_P60472 | 0.42591448 | 0.048110078 |
| TFDP1 | A_24_P483083 | 0.425916901 | 0.048108669 |
| HOXA13 | A_23_P31306 | 0.425936917 | 0.048097028 |
| PON2 | A_23_P31399 | 0.426027547 | 0.048044347 |
| CRB1 | A_24_P196528 | 0.426065465 | 0.04802232 |
| RBM12B | A_32_P482979 | 0.426113745 | 0.047994284 |
| UBA1 | A_24_P237374 | 0.426120393 | 0.047990425 |
| HNRNPC | A_24_P178423 | 0.426169382 | 0.047961991 |
| MTHFSD | A_23_P100455 | 0.426221006 | 0.047932043 |
| MTHFD2L | A_23_P112846 | 0.426464164 | 0.047791178 |
| RPS10 | A_32_P14894 | 0.426653372 | 0.047681788 |
| ZNF253 | A_24_P22981 | 0.426674683 | 0.04766948 |
| DUSP18 | A_23_P143650 | 0.426769852 | 0.047614542 |
| UBXN11 | A_24_P239811 | 0.426851797 | 0.047567279 |
| BDH2 | A_24_P80500 | 0.426918721 | 0.047528706 |
| RUNX3 | A_23_P51231 | 0.426997378 | 0.0474834 |
| TTLL4 | A_23_P142697 | 0.427072048 | 0.047440423 |
| KIAA0776 | A_23_P30956 | 0.427328399 | 0.047293106 |
| MICAL3 | A_24_P366082 | 0.427376416 | 0.047265552 |
| PHF10 | A_23_P503200 | 0.427391365 | 0.047256975 |
| DAD1 | A_24_P47870 | 0.427438931 | 0.047229696 |
| SNRPC | A_24_P83922 | 0.427493803 | 0.047198241 |
| SERPINI2 | A_23_P132826 | 0.427566059 | 0.047156847 |
| SLC22A3 | A_23_P19733 | 0.427802063 | 0.047021839 |
| UTP15 | A_23_P213441 | 0.427876537 | 0.046979298 |
| JARID2 | A_23_P214876 | 0.427948055 | 0.046938473 |
| HAUS6 | A_23_P146347 | 0.428197632 | 0.046796223 |
| PPP2R3C | A_23_P88201 | 0.428201065 | 0.046794269 |
| DTWD1 | A_24_P70117 | 0.428221086 | 0.046782872 |
| DERL2 | A_23_P118749 | 0.428254629 | 0.046763784 |
| BMP2K | A_24_P86240 | 0.428465219 | 0.046644079 |
| DUSP5P | A_24_P367602 | 0.428480663 | 0.04663531 |
| DHRSX | A_24_P378987 | 0.428530527 | 0.046607005 |
| FAM35B | A_32_P166598 | 0.428608699 | 0.046562658 |
| SPIN3 | A_24_P136725 | 0.428632098 | 0.046549391 |
| PSMA7 | A_23_P91468 | 0.42878039 | 0.046465373 |
| C2CD3 | A_23_P150741 | 0.428976222 | 0.046354602 |
| CRNDE | A_32_P104063 | 0.429010044 | 0.046335491 |
| HOXA13 | A_23_P389281 | 0.429046586 | 0.046314851 |
| THADA | A_23_P108376 | 0.42914834 | 0.046257413 |
| MUS81 | A_24_P412238 | 0.429195365 | 0.046230888 |
| SLC25A30 | A_23_P159284 | 0.429229673 | 0.046211543 |
| TTC27 | A_23_P131227 | 0.429240158 | 0.046205632 |
| PRAME | A_23_P166360 | 0.429248595 | 0.046200877 |
| TDH | A_23_P337778 | 0.429252167 | 0.046198863 |
| CDK6 | A_24_P166663 | 0.429440231 | 0.046092956 |
| NUP50 | A_23_P434768 | 0.429467214 | 0.046077776 |
| RPL13AP3 | A_24_P272873 | 0.429532883 | 0.046040849 |
| ALDH9A1 | A_24_P385280 | 0.429621501 | 0.045991053 |
| RANGRF | A_23_P55136 | 0.429628562 | 0.045987088 |
| RING1 | A_24_P200694 | 0.429655763 | 0.045971812 |
| GRAMD3 | A_23_P22350 | 0.429656131 | 0.045971605 |
| SMAD5 | A_23_P144944 | 0.429662748 | 0.045967891 |
| TMEM33 | A_23_P213069 | 0.429859355 | 0.045857607 |
| WDR12 | A_23_P28625 | 0.429909579 | 0.045829467 |
| RREB1 | A_23_P325411 | 0.429920668 | 0.045823256 |
| SMAD4 | A_23_P27346 | 0.43006534 | 0.045742283 |
| GALNT1 | A_23_P306105 | 0.430261923 | 0.045632434 |
| GORAB | A_23_P368145 | 0.430596799 | 0.045445779 |
| EVI5 | A_24_P96593 | 0.430769382 | 0.045349816 |
| NOTCH1 | A_23_P60387 | 0.430876531 | 0.045290316 |
| TFB2M | A_23_P45940 | 0.430876783 | 0.045290177 |
| C11orf57 | A_24_P21727 | 0.430888649 | 0.045283591 |
| C2orf42 | A_23_P79441 | 0.430924289 | 0.045263816 |
| BRCA2 | A_23_P99452 | 0.431009912 | 0.045216334 |
| PRPF3 | A_23_P97573 | 0.431032765 | 0.045203668 |
| DISP1 | A_23_P201319 | 0.431078861 | 0.045178127 |
| RNF138 | A_24_P202512 | 0.431367584 | 0.04501841 |
| XPO5 | A_23_P256855 | 0.431515733 | 0.044936626 |
| ZNF254 | A_23_P324453 | 0.431559109 | 0.044912703 |
| KIF9 | A_23_P212307 | 0.431604929 | 0.044887443 |
| FAM83D | A_23_P323751 | 0.431608204 | 0.044885638 |
| RLN2 | A_23_P216455 | 0.431623909 | 0.044876983 |
| IGSF9 | A_23_P85441 | 0.431707734 | 0.044830807 |
| KIAA0406 | A_23_P109001 | 0.43171915 | 0.044824521 |
| DDX19A | A_23_P129606 | 0.431753595 | 0.04480556 |
| DPY19L1P1 | A_24_P652033 | 0.432076366 | 0.044628183 |
| B3GALNT2 | A_24_P212531 | 0.432125248 | 0.044601367 |
| P4HA1 | A_24_P406693 | 0.432138467 | 0.044594118 |
| PTPLAD1 | A_23_P99927 | 0.432149877 | 0.044587861 |
| TIGD6 | A_24_P337419 | 0.432191397 | 0.0445651 |
| TAF1 | A_23_P11237 | 0.432250052 | 0.044532961 |
| SOX1 | A_23_P434416 | 0.432283257 | 0.044514774 |
| PHF2 | A_23_P502376 | 0.432353732 | 0.044476194 |
| RFX4 | A_23_P76332 | 0.43247238 | 0.044411301 |
| RPL34 | A_24_P188878 | 0.43247414 | 0.044410339 |
| C6orf203 | A_23_P259333 | 0.432481188 | 0.044406487 |
| FMN2 | A_23_P342869 | 0.432562658 | 0.044361975 |
| BCL2L12 | A_23_P50477 | 0.432615533 | 0.044333104 |
| HAUS6 | A_24_P226949 | 0.43265606 | 0.044310986 |
| ZNF135 | A_23_P348227 | 0.432683513 | 0.044296008 |
| NETO2 | A_32_P77989 | 0.432806 | 0.044229228 |
| RPS5 | A_24_P41180 | 0.432976648 | 0.044136322 |
| ANAPC4 | A_23_P317800 | 0.433027175 | 0.044108842 |
| TTTY11 | A_23_P160017 | 0.433155458 | 0.044039135 |
| HAUS1 | A_23_P413796 | 0.433285592 | 0.043968508 |
| RPS5 | A_23_P208265 | 0.433377779 | 0.043918531 |
| LRRC48 | A_23_P255701 | 0.433540862 | 0.043830226 |
| C3orf58 | A_23_P144134 | 0.433568884 | 0.043815066 |
| ARL8A | A_23_P415033 | 0.433759759 | 0.043711916 |
| PPP1R13L | A_23_P119095 | 0.433788619 | 0.043696336 |
| NDUFB11 | A_23_P22765 | 0.434014153 | 0.043574734 |
| OSTCL | A_23_P304395 | 0.434019177 | 0.043572028 |
| ELP2 | A_23_P78444 | 0.434029684 | 0.04356637 |
| KIAA0174 | A_24_P339157 | 0.434046627 | 0.043557246 |
| LOC284900 | A_32_P461976 | 0.434052763 | 0.043553942 |
| SDCCAG8 | A_23_P34546 | 0.434095555 | 0.043530908 |
| CHEK1 | A_23_P116123 | 0.43414851 | 0.043502417 |
| HEPACAM | A_24_P213950 | 0.434235402 | 0.043455697 |
| FIP1L1 | A_23_P58337 | 0.434299898 | 0.043421045 |
| WHSC1 | A_24_P2463 | 0.434520532 | 0.043302666 |
| SYNCRIP | A_24_P940599 | 0.434553031 | 0.04328525 |
| ZNF333 | A_23_P5221 | 0.43467476 | 0.043220066 |
| ZNF135 | A_32_P224302 | 0.434676722 | 0.043219016 |
| IQCG | A_23_P300076 | 0.434714254 | 0.043198934 |
| LIX1L | A_24_P687594 | 0.434734195 | 0.043188267 |
| ZNF433 | A_23_P27649 | 0.434884812 | 0.043107765 |
| PPPDE1 | A_23_P201445 | 0.43488903 | 0.043105513 |
| LYPLA1 | A_23_P19192 | 0.434976594 | 0.043058767 |
| CTNND1 | A_23_P251316 | 0.435211249 | 0.042933693 |
| MCART1 | A_24_P501520 | 0.435501474 | 0.042779391 |
| SLC2A4RG | A_23_P102575 | 0.435518645 | 0.042770276 |
| MRPS31 | A_23_P162807 | 0.435564193 | 0.042746103 |
| TET1 | A_32_P202134 | 0.435597715 | 0.042728319 |
| PTGR2 | A_23_P48705 | 0.435687875 | 0.042680517 |
| IFT88 | A_23_P48339 | 0.435702364 | 0.042672839 |
| FOXRED2 | A_23_P369948 | 0.435710445 | 0.042668557 |
| FGF17 | A_23_P429363 | 0.435828045 | 0.042606283 |
| ZNF473 | A_23_P107942 | 0.435838772 | 0.042600606 |
| ZNF229 | A_23_P130509 | 0.43583983 | 0.042600046 |
| CEP350 | A_23_P201816 | 0.435894461 | 0.042571144 |
| DONSON | A_23_P425502 | 0.435911509 | 0.042562128 |
| H3F3A | A_24_P323835 | 0.436028327 | 0.042500388 |
| PLGLB1 | A_32_P6535 | 0.436043164 | 0.042492551 |
| C16orf55 | A_24_P159335 | 0.436159865 | 0.042430951 |
| CRLS1 | A_23_P400435 | 0.43626339 | 0.042376365 |
| TAF1B | A_23_P165891 | 0.436277402 | 0.042368981 |
| ZNFX1 | A_24_P23034 | 0.43638526 | 0.042312176 |
| DDX20 | A_23_P63153 | 0.436393573 | 0.0423078 |
| AEBP2 | A_24_P18621 | 0.436693142 | 0.042150352 |
| SNRPG | A_23_P102235 | 0.43669903 | 0.042147262 |
| ZNF45 | A_23_P426472 | 0.436814041 | 0.042086939 |
| INTS8 | A_23_P43079 | 0.436850253 | 0.04206796 |
| SYCP2 | A_23_P210675 | 0.437008817 | 0.041984933 |
| POLR1C | A_23_P70409 | 0.437083371 | 0.04194594 |
| QRSL1 | A_24_P163477 | 0.437201364 | 0.041884285 |
| SURF6 | A_24_P21447 | 0.437212294 | 0.041878577 |
| MELK | A_23_P94422 | 0.437220353 | 0.041874369 |
| RFX4 | A_23_P204342 | 0.437334378 | 0.041814865 |
| PDCL | A_23_P349882 | 0.437336857 | 0.041813572 |
| RCOR3 | A_24_P276816 | 0.43741191 | 0.041774443 |
| ZNF77 | A_32_P182394 | 0.437435004 | 0.041762409 |
| ZNF773 | A_23_P153256 | 0.437543855 | 0.041705722 |
| HOXA2 | A_24_P200854 | 0.437614757 | 0.041668831 |
| DNTTIP1 | A_23_P131816 | 0.43762236 | 0.041664876 |
| TET1 | A_23_P322756 | 0.437987763 | 0.041475169 |
| ADRB2 | A_23_P145024 | 0.438058348 | 0.041438601 |
| TMEM231 | A_23_P359497 | 0.438188022 | 0.041371487 |
| RIT1 | A_23_P316601 | 0.438224558 | 0.041352593 |
| GNPDA1 | A_23_P144622 | 0.438339283 | 0.041293307 |
| MRPL24 | A_23_P137848 | 0.438371615 | 0.041276611 |
| FAM69C | A_32_P203404 | 0.43839223 | 0.041265969 |
| DSN1 | A_23_P165937 | 0.438415449 | 0.041253984 |
| TIGD1 | A_23_P120170 | 0.438441426 | 0.041240579 |
| ZMYM2 | A_23_P99405 | 0.438492872 | 0.041214042 |
| FERMT2 | A_23_P88347 | 0.438506892 | 0.041206812 |
| ANXA4 | A_23_P16976 | 0.438820169 | 0.041045525 |
| AKR7L | A_23_P320304 | 0.438840938 | 0.04103485 |
| RBM23 | A_24_P207091 | 0.4388649 | 0.041022536 |
| TTC30A | A_23_P131611 | 0.439356555 | 0.040770518 |
| CXCL5 | A_24_P277367 | 0.439358719 | 0.040769412 |
| LOC400891 | A_23_P80200 | 0.439519339 | 0.040687344 |
| FBXO28 | A_23_P137578 | 0.43964464 | 0.040623412 |
| FAM35A | A_32_P180210 | 0.439916691 | 0.040484874 |
| EXO1 | A_23_P23303 | 0.439978974 | 0.040453209 |
| MRPL19 | A_23_P102262 | 0.440023037 | 0.040430819 |
| HSD17B10 | A_23_P45396 | 0.440165275 | 0.040358609 |
| FASTKD2 | A_23_P209649 | 0.440277311 | 0.040301803 |
| FAM160B2 | A_23_P157620 | 0.440302095 | 0.040289244 |
| TAF1 | A_32_P192615 | 0.440312298 | 0.040284076 |
| LOC644563 | A_24_P127121 | 0.440316192 | 0.040282103 |
| ARHGAP11A | A_23_P136805 | 0.440374323 | 0.040252664 |
| TMEM11 | A_23_P107036 | 0.44043837 | 0.040220249 |
| NMT2 | A_23_P138693 | 0.440477998 | 0.040200203 |
| SF3B14 | A_23_P165402 | 0.440480315 | 0.040199032 |
| RPGRIP1L | A_32_P123966 | 0.440584195 | 0.040146521 |
| NSL1 | A_24_P370970 | 0.440625707 | 0.040125552 |
| FAM36A | A_24_P79712 | 0.440687553 | 0.040094328 |
| SMC4 | A_23_P91900 | 0.440890833 | 0.039991832 |
| ARMC4 | A_32_P141418 | 0.440895061 | 0.039989703 |
| CEP110 | A_23_P43580 | 0.44118795 | 0.039842391 |
| CYP4F2 | A_24_P168494 | 0.441203389 | 0.039834638 |
| LOC644950 | A_24_P229756 | 0.441624457 | 0.039623634 |
| C7orf57 | A_32_P3955 | 0.441633222 | 0.039619251 |
| ST13 | A_32_P156892 | 0.441685299 | 0.039593218 |
| C14orf33 | A_32_P137399 | 0.441690555 | 0.039590591 |
| PUM2 | A_24_P2995 | 0.441707768 | 0.039581989 |
| ROCK1 | A_24_P538403 | 0.441744355 | 0.039563711 |
| KHDRBS1 | A_23_P200386 | 0.441782698 | 0.039544564 |
| MTX1 | A_23_P137865 | 0.442017549 | 0.03942744 |
| SLC30A1 | A_24_P937095 | 0.44208385 | 0.039394423 |
| SFI1 | A_24_P242021 | 0.442216411 | 0.039328476 |
| MAP4K3 | A_24_P285522 | 0.44226749 | 0.039303089 |
| C6orf70 | A_32_P129530 | 0.442286436 | 0.039293675 |
| ZNF559 | A_24_P284584 | 0.442490354 | 0.039192468 |
| MRPS16 | A_24_P12932 | 0.442587789 | 0.039144182 |
| RFX3 | A_23_P9289 | 0.442738939 | 0.039069369 |
| FBXL5 | A_23_P213247 | 0.44274308 | 0.039067321 |
| FRMD4A | A_24_P942321 | 0.442819469 | 0.039029555 |
| RAB5C | A_23_P107214 | 0.443095259 | 0.038893447 |
| KDM5C | A_23_P62270 | 0.443277079 | 0.038803918 |
| C16orf48 | A_23_P14975 | 0.443445274 | 0.038721242 |
| ANP32E | A_24_P225468 | 0.443635795 | 0.038627759 |
| NUDCD2 | A_23_P360245 | 0.443777605 | 0.038558292 |
| THAP7 | A_23_P109410 | 0.443796614 | 0.038548987 |
| RG9MTD3 | A_23_P400459 | 0.443888866 | 0.038503857 |
| DDX19B | A_24_P407704 | 0.443922412 | 0.038487457 |
| FAM90A7 | A_24_P515319 | 0.444013577 | 0.038442915 |
| TROVE2 | A_23_P200767 | 0.444037082 | 0.038431437 |
| RAB4A | A_23_P369919 | 0.444059436 | 0.038420524 |
| C12orf35 | A_24_P273561 | 0.444081198 | 0.038409902 |
| ZNF354C | A_23_P424126 | 0.44426918 | 0.038318245 |
| ANAPC10 | A_23_P250994 | 0.444409452 | 0.038249963 |
| RAB9A | A_23_P45389 | 0.444473698 | 0.03821872 |
| RUNDC2C | A_32_P64016 | 0.444531363 | 0.038190696 |
| CHD9 | A_24_P942250 | 0.444590687 | 0.038161881 |
| TMEM99 | A_32_P32835 | 0.444667491 | 0.038124602 |
| CENPL | A_23_P126120 | 0.444717991 | 0.038100106 |
| HHEX | A_23_P47034 | 0.444731198 | 0.038093701 |
| ZSWIM1 | A_23_P5974 | 0.4447574 | 0.038080998 |
| IFT140 | A_23_P140725 | 0.445186587 | 0.03787339 |
| FAM135A | A_23_P58862 | 0.445413553 | 0.03776396 |
| NEK11 | A_23_P211973 | 0.445421794 | 0.037759991 |
| YY1AP1 | A_23_P103661 | 0.445566407 | 0.037690403 |
| TMCO1 | A_24_P276583 | 0.445709906 | 0.03762145 |
| CLK2 | A_23_P85888 | 0.445746959 | 0.037603661 |
| CASP8AP2 | A_23_P58898 | 0.445856214 | 0.037551249 |
| ZNF177 | A_23_P164706 | 0.445938195 | 0.037511958 |
| ITPKB | A_23_P372255 | 0.446131602 | 0.037419392 |
| DTL | A_23_P10385 | 0.446154899 | 0.037408254 |
| AKR1A1 | A_24_P79755 | 0.446357616 | 0.037311447 |
| IPO9 | A_23_P97157 | 0.446445875 | 0.037269361 |
| ZNF578 | A_23_P339601 | 0.446581741 | 0.037204646 |
| NFIA | A_32_P155030 | 0.446625222 | 0.037183954 |
| SOX9 | A_23_P26847 | 0.446758012 | 0.037120817 |
| CDC26 | A_32_P228501 | 0.447362954 | 0.036834253 |
| UBE2CBP | A_24_P626931 | 0.447662472 | 0.036693015 |
| TRMT2A | A_24_P305662 | 0.447696291 | 0.036677094 |
| PLGLB1 | A_32_P220750 | 0.447797637 | 0.036629416 |
| SAP130 | A_23_P154188 | 0.447856858 | 0.036601579 |
| TOP2A | A_23_P118834 | 0.447965038 | 0.036550771 |
| NCAPD2 | A_23_P25293 | 0.448047211 | 0.036512214 |
| TAF4 | A_23_P80062 | 0.448121302 | 0.036477477 |
| ECT2 | A_23_P9574 | 0.448317693 | 0.036385525 |
| IGSF6 | A_23_P106629 | 0.448324575 | 0.036382307 |
| ADAL | A_24_P193482 | 0.448764575 | 0.036176972 |
| CDT1 | A_24_P176374 | 0.448914765 | 0.036107092 |
| LRIG3 | A_23_P47885 | 0.448930633 | 0.036099715 |
| CCDC120 | A_23_P73540 | 0.449078121 | 0.036031206 |
| PHF20 | A_24_P181944 | 0.449181836 | 0.035983092 |
| SRRT | A_23_P111487 | 0.449266856 | 0.035943688 |
| WDR26 | A_24_P42803 | 0.449395112 | 0.035884309 |
| NR3C1 | A_24_P214754 | 0.449448212 | 0.035859748 |
| FLJ26850 | A_32_P215100 | 0.449455309 | 0.035856466 |
| XRCC2 | A_32_P77343 | 0.449479978 | 0.035845061 |
| C1orf112 | A_23_P11862 | 0.449554576 | 0.035810589 |
| WNK3 | A_32_P178879 | 0.449563466 | 0.035806483 |
| ASPM | A_23_P52017 | 0.449625795 | 0.035777704 |
| WIPF2 | A_23_P366455 | 0.449708225 | 0.035739671 |
| ZFP36L1 | A_23_P99540 | 0.450385048 | 0.035428589 |
| PTGR2 | A_23_P48713 | 0.450675416 | 0.035295784 |
| DNAJC18 | A_23_P123987 | 0.450679734 | 0.035293812 |
| TATDN1 | A_32_P224911 | 0.450952767 | 0.0351693 |
| ETHE1 | A_23_P142294 | 0.450956399 | 0.035167646 |
| TRDMT1 | A_24_P252705 | 0.45099961 | 0.035147972 |
| PARP16 | A_23_P163278 | 0.451144155 | 0.035082226 |
| OR1L4 | A_23_P83244 | 0.451296086 | 0.035013224 |
| NFIA | A_32_P155026 | 0.451505016 | 0.03491851 |
| ZBTB25 | A_23_P48628 | 0.451548655 | 0.034898753 |
| NDC80 | A_23_P50108 | 0.451552987 | 0.034896793 |
| RBM23 | A_24_P219156 | 0.451639142 | 0.034857814 |
| DDX31 | A_24_P112377 | 0.45184673 | 0.034764037 |
| CD300LF | A_23_P55020 | 0.451898982 | 0.034740464 |
| ZNF140 | A_23_P150841 | 0.451973074 | 0.034707059 |
| ZC3H11A | A_23_P58967 | 0.452068608 | 0.034664024 |
| EXT1 | A_32_P226556 | 0.452138573 | 0.034632534 |
| PHKB | A_23_P206532 | 0.452186081 | 0.034611164 |
| LRP5L | A_24_P387514 | 0.452290934 | 0.034564037 |
| COLEC11 | A_23_P120125 | 0.452364355 | 0.034531067 |
| LOC100129502 | A_24_P196384 | 0.452372503 | 0.034527409 |
| CNOT3 | A_23_P38987 | 0.452389173 | 0.034519928 |
| MUDENG | A_23_P37265 | 0.452768455 | 0.034350048 |
| ZUFSP | A_32_P109522 | 0.452943582 | 0.034271831 |
| HDGF | A_24_P376707 | 0.453143077 | 0.034182902 |
| GGPS1 | A_23_P52189 | 0.453335964 | 0.034097091 |
| HNRNPL | A_32_P109572 | 0.4534076 | 0.034065265 |
| PROCA1 | A_23_P427148 | 0.453505627 | 0.034021753 |
| RFWD2 | A_23_P396194 | 0.453654745 | 0.033955646 |
| FAM81B | A_23_P360354 | 0.453684697 | 0.03394238 |
| KIF27 | A_23_P71741 | 0.453727051 | 0.033923627 |
| ERCC6L | A_23_P96325 | 0.453739406 | 0.033918159 |
| RNU12 | A_24_P23445 | 0.453898706 | 0.033847711 |
| PMF1 | A_23_P635 | 0.453979665 | 0.033811953 |
| C5orf51 | A_32_P6832 | 0.454046155 | 0.033782608 |
| MTPAP | A_23_P1145 | 0.454165839 | 0.033729835 |
| PRC1 | A_23_P206059 | 0.454195297 | 0.033716857 |
| ZNF404 | A_23_P90333 | 0.454279989 | 0.033679564 |
| FAM123B | A_23_P308150 | 0.454487025 | 0.033588536 |
| CCDC49 | A_24_P48318 | 0.454519677 | 0.033574198 |
| TRIM39 | A_24_P89413 | 0.454610054 | 0.033534536 |
| DIS3 | A_24_P395317 | 0.454666088 | 0.033509965 |
| PIPOX | A_23_P164258 | 0.454751414 | 0.033472575 |
| ELP4 | A_23_P24623 | 0.454909074 | 0.033403575 |
| SYPL1 | A_24_P141005 | 0.455015476 | 0.033357072 |
| INTS2 | A_23_P420269 | 0.45505476 | 0.033339916 |
| SLC37A2 | A_23_P86931 | 0.455060292 | 0.0333375 |
| C8orf58 | A_23_P310483 | 0.4550939 | 0.033322829 |
| KATNA1 | A_23_P113803 | 0.455370694 | 0.033202193 |
| C19orf18 | A_23_P303803 | 0.455611318 | 0.033097601 |
| ETAA1 | A_23_P51117 | 0.455613146 | 0.033096807 |
| RHBDD3 | A_23_P155009 | 0.45568604 | 0.033065174 |
| FBXO30 | A_23_P256231 | 0.45584744 | 0.032995218 |
| CRY1 | A_24_P407235 | 0.45612195 | 0.032876505 |
| ARL6IP6 | A_23_P28169 | 0.456133807 | 0.032871385 |
| C21orf59 | A_23_P91491 | 0.45616137 | 0.032859486 |
| CGRRF1 | A_23_P37283 | 0.45618766 | 0.032848139 |
| FGF7 | A_23_P14612 | 0.456191875 | 0.03284632 |
| CTNND1 | A_24_P38930 | 0.456216487 | 0.032835701 |
| IRX1 | A_23_P133457 | 0.456291964 | 0.032803151 |
| STX6 | A_23_P331992 | 0.456311548 | 0.03279471 |
| HEATR5A | A_23_P433369 | 0.456481178 | 0.032721665 |
| CDC14C | A_23_P367043 | 0.456484024 | 0.032720441 |
| C5orf33 | A_23_P337168 | 0.45664897 | 0.032649538 |
| RIBC1 | A_23_P355510 | 0.456741489 | 0.032609822 |
| LOC100129616 | A_23_P372516 | 0.456976741 | 0.032509005 |
| FAM36A | A_32_P106646 | 0.457174725 | 0.03242435 |
| S100PBP | A_23_P338952 | 0.457179144 | 0.032422463 |
| ZNF395 | A_23_P146077 | 0.457462883 | 0.03230145 |
| PRDM13 | A_23_P256581 | 0.457645204 | 0.03222388 |
| CNOT1 | A_24_P193570 | 0.457699825 | 0.032200669 |
| HNRNPL | A_24_P42122 | 0.457731582 | 0.032187181 |
| ZBTB5 | A_23_P216476 | 0.457745654 | 0.032181205 |
| RABEPK | A_24_P386323 | 0.457752145 | 0.032178449 |
| 1-Sep | A_23_P21057 | 0.457752829 | 0.032178159 |
| ZNF500 | A_24_P126305 | 0.457796611 | 0.032159574 |
| SF3B1 | A_32_P31633 | 0.457865762 | 0.032130238 |
| TDRD3 | A_32_P187617 | 0.457983726 | 0.032080242 |
| POLD1 | A_23_P50456 | 0.45799409 | 0.032075853 |
| MRPL50 | A_23_P253412 | 0.458030319 | 0.032060512 |
| BMPR1B | A_32_P217901 | 0.458037821 | 0.032057336 |
| ACOX1 | A_23_P77761 | 0.458137881 | 0.032015001 |
| WDR26 | A_23_P85604 | 0.458240989 | 0.031971422 |
| PCDH18 | A_24_P284959 | 0.458306537 | 0.031943743 |
| FDXACB1 | A_23_P362261 | 0.458320888 | 0.031937685 |
| MCM6 | A_23_P90612 | 0.458328299 | 0.031934558 |
| HIST2H2AB | A_23_P343927 | 0.458336253 | 0.031931201 |
| C7orf36 | A_23_P403521 | 0.458360675 | 0.031920896 |
| PI4KB | A_24_P222860 | 0.458397953 | 0.031905172 |
| ABL1 | A_24_P282416 | 0.458510745 | 0.031857632 |
| FANCD2 | A_23_P345678 | 0.458720716 | 0.031769282 |
| AIDA | A_23_P97457 | 0.458778587 | 0.031744965 |
| TFAP2B | A_24_P20954 | 0.458834541 | 0.031721468 |
| DDAH1 | A_23_P201386 | 0.459068882 | 0.03162321 |
| OSTC | A_23_P411814 | 0.45920531 | 0.031566117 |
| GABPA | A_23_P166219 | 0.459241465 | 0.031551001 |
| PIGO | A_23_P60240 | 0.459436912 | 0.031469382 |
| ERAL1 | A_23_P71981 | 0.459577994 | 0.03141057 |
| C10orf57 | A_23_P97853 | 0.460217052 | 0.031145259 |
| SKA2 | A_32_P164522 | 0.460363846 | 0.031084567 |
| LRRC58 | A_24_P23865 | 0.460386101 | 0.031075374 |
| HPGDS | A_23_P10506 | 0.460426528 | 0.031058679 |
| VPS72 | A_23_P115215 | 0.460482105 | 0.03103574 |
| C14orf145 | A_23_P430201 | 0.460525347 | 0.031017902 |
| ALG10B | A_32_P103309 | 0.460607769 | 0.030983924 |
| WDR34 | A_23_P20823 | 0.460685441 | 0.03095193 |
| MAD2L1 | A_23_P92441 | 0.46072057 | 0.030937469 |
| JTB | A_23_P34983 | 0.460960934 | 0.030838664 |
| TMCO1 | A_23_P355067 | 0.461070585 | 0.030793674 |
| HOXC9 | A_23_P25150 | 0.461086774 | 0.030787036 |
| FAM120B | A_24_P346181 | 0.461127684 | 0.030770267 |
| FAM165B | A_24_P74487 | 0.461216385 | 0.030733932 |
| ALDH3A1 | A_23_P207213 | 0.461379038 | 0.030667393 |
| COQ7 | A_24_P398972 | 0.461544484 | 0.030599828 |
| SHPRH | A_23_P337790 | 0.46163378 | 0.03056341 |
| C18orf25 | A_23_P345361 | 0.461739843 | 0.030520199 |
| WDHD1 | A_23_P25873 | 0.461743506 | 0.030518707 |
| ZNF223 | A_23_P108129 | 0.461787159 | 0.030500937 |
| BUB1 | A_23_P124417 | 0.461978104 | 0.030423305 |
| ZNF582 | A_23_P395464 | 0.462087836 | 0.030378762 |
| PALB2 | A_23_P129569 | 0.462209901 | 0.030329274 |
| CDC14B | A_23_P20622 | 0.462444008 | 0.030234539 |
| GON4L | A_24_P212457 | 0.462561005 | 0.030187283 |
| SRP9 | A_32_P151594 | 0.462875182 | 0.030060673 |
| CDX2 | A_23_P76654 | 0.462876047 | 0.030060325 |
| GM2A | A_24_P925314 | 0.462931335 | 0.030038088 |
| ZNF420 | A_23_P380951 | 0.463073798 | 0.02998085 |
| C10orf79 | A_23_P333038 | 0.463231089 | 0.029917755 |
| ZNF879 | A_24_P791669 | 0.463416743 | 0.029843417 |
| ZNF271 | A_23_P147199 | 0.463456299 | 0.029827598 |
| PPARD | A_24_P252130 | 0.463633853 | 0.02975667 |
| SF3A1 | A_23_P104676 | 0.463684848 | 0.029736324 |
| NDC80 | A_24_P14156 | 0.463759628 | 0.029706508 |
| C1orf51 | A_24_P114103 | 0.464090564 | 0.029574842 |
| WHSC1 | A_23_P389919 | 0.464330657 | 0.029479609 |
| DARS2 | A_23_P148984 | 0.464566643 | 0.029386243 |
| FASTKD5 | A_23_P380724 | 0.464903327 | 0.029253442 |
| BAG2 | A_23_P356554 | 0.464926899 | 0.029244161 |
| TIPRL | A_32_P229065 | 0.464952714 | 0.029234001 |
| ZNF701 | A_23_P130444 | 0.465118896 | 0.029168663 |
| C6orf167 | A_24_P83678 | 0.465564089 | 0.028994195 |
| C14orf109 | A_23_P140154 | 0.465626318 | 0.028969874 |
| DDX46 | A_32_P105195 | 0.465906255 | 0.028860667 |
| ASPM | A_24_P911179 | 0.46592843 | 0.02885203 |
| FUT10 | A_23_P22409 | 0.46595035 | 0.028843494 |
| PMS2 | A_23_P93792 | 0.466021168 | 0.028815932 |
| HMGB2 | A_23_P155765 | 0.466216817 | 0.028739894 |
| NFIA | A_23_P85682 | 0.466253431 | 0.028725682 |
| PLCD3 | A_23_P351757 | 0.466284111 | 0.028713777 |
| RP5-1022P6.2 | A_23_P353704 | 0.466294931 | 0.02870958 |
| XPR1 | A_23_P63534 | 0.466442635 | 0.028652329 |
| RFX4 | A_24_P224158 | 0.466580843 | 0.028598841 |
| COLEC11 | A_24_P388322 | 0.466668372 | 0.028565008 |
| C19orf52 | A_23_P310532 | 0.466748672 | 0.028533997 |
| ZNF615 | A_24_P190345 | 0.466827442 | 0.028503602 |
| ZNF124 | A_23_P52176 | 0.466942034 | 0.028459431 |
| RFXAP | A_23_P315789 | 0.467011974 | 0.028432498 |
| TMEM14C | A_23_P111288 | 0.467022638 | 0.028428394 |
| C11orf48 | A_23_P1948 | 0.467068782 | 0.028410638 |
| RPS10 | A_23_P318646 | 0.467274118 | 0.028331732 |
| APH1A | A_23_P12329 | 0.467443172 | 0.028266899 |
| TMEM81 | A_23_P46690 | 0.467505907 | 0.02824287 |
| WDR19 | A_24_P394368 | 0.467575158 | 0.028216364 |
| C22orf39 | A_23_P388244 | 0.467696543 | 0.028169951 |
| C13orf34 | A_23_P25626 | 0.467717433 | 0.02816197 |
| PCDHGB4 | A_23_P359588 | 0.467794797 | 0.028132427 |
| CBY1 | A_24_P74571 | 0.468030371 | 0.028042622 |
| CCDC88C | A_23_P379945 | 0.468261105 | 0.027954882 |
| PEX19 | A_23_P160188 | 0.468391979 | 0.027905212 |
| SNRPB2 | A_24_P194260 | 0.468434453 | 0.027889107 |
| FLJ31306 | A_24_P495122 | 0.468739213 | 0.027773769 |
| SPAG9 | A_24_P365025 | 0.469116172 | 0.027631631 |
| C1orf194 | A_32_P15512 | 0.469181119 | 0.0276072 |
| TSPAN16 | A_23_P142070 | 0.469211871 | 0.027595638 |
| FANCD2 | A_32_P24165 | 0.469231526 | 0.027588251 |
| ZNF28 | A_24_P282043 | 0.46925894 | 0.02757795 |
| CHD1L | A_23_P45831 | 0.469305636 | 0.02756041 |
| NPL | A_23_P381431 | 0.469355062 | 0.027541854 |
| GPS2 | A_23_P218405 | 0.469404097 | 0.027523455 |
| KIAA1383 | A_23_P413021 | 0.469497435 | 0.027488459 |
| EVI5 | A_23_P34942 | 0.469514458 | 0.02748208 |
| LOC401588 | A_24_P256654 | 0.469608319 | 0.027446931 |
| SALL3 | A_23_P393670 | 0.469768479 | 0.027387036 |
| GTF2A2 | A_24_P270525 | 0.469976824 | 0.027309277 |
| PPP2R5A | A_24_P251221 | 0.470386367 | 0.027156938 |
| ACOX1 | A_23_P206945 | 0.470478887 | 0.027122617 |
| ZMYND11 | A_24_P201323 | 0.470806568 | 0.027001338 |
| LQK1 | A_32_P190404 | 0.47084622 | 0.026986692 |
| PGK2 | A_23_P331063 | 0.470948248 | 0.026949035 |
| TTC25 | A_23_P73150 | 0.471127702 | 0.026882902 |
| C8orf37 | A_24_P237566 | 0.471185112 | 0.026861772 |
| TDH | A_23_P337785 | 0.471246768 | 0.026839095 |
| RNF5 | A_23_P8095 | 0.471253658 | 0.026836561 |
| POGZ | A_23_P500734 | 0.471292496 | 0.026822285 |
| ZNF560 | A_23_P368773 | 0.471401195 | 0.026782362 |
| FANCF | A_23_P12896 | 0.471533397 | 0.02673387 |
| ZSWIM1 | A_23_P5976 | 0.472023031 | 0.02655488 |
| HNRNPF | A_23_P75330 | 0.472373163 | 0.026427472 |
| C11orf93 | A_23_P116173 | 0.472394973 | 0.026419552 |
| FANCG | A_23_P71644 | 0.472440024 | 0.026403198 |
| RPRD1B | A_24_P250499 | 0.472747968 | 0.026291627 |
| IRF2BP2 | A_24_P497186 | 0.473172244 | 0.026138522 |
| UXT | A_23_P159663 | 0.473245735 | 0.026112075 |
| C20orf96 | A_23_P328034 | 0.473300798 | 0.026092273 |
| KCNJ8 | A_23_P64879 | 0.473336235 | 0.026079535 |
| TFF3 | A_24_P289208 | 0.473343676 | 0.026076861 |
| RRP15 | A_23_P23356 | 0.473615318 | 0.025979393 |
| DHX35 | A_23_P5945 | 0.473742755 | 0.025933768 |
| CD99 | A_23_P217510 | 0.474206126 | 0.025768409 |
| TCF7 | A_23_P7582 | 0.47428323 | 0.025740975 |
| ECT2 | A_24_P366033 | 0.474333503 | 0.0257231 |
| ZNF430 | A_23_P56298 | 0.47434491 | 0.025719046 |
| INTS7 | A_23_P52082 | 0.474519168 | 0.025657173 |
| CCDC121 | A_23_P5742 | 0.475085837 | 0.025456786 |
| MIPEP | A_23_P117274 | 0.475135963 | 0.025439121 |
| EED | A_23_P53217 | 0.475471678 | 0.025321061 |
| PCGF6 | A_23_P115703 | 0.475694301 | 0.025243013 |
| LGR4 | A_32_P17765 | 0.475702235 | 0.025240235 |
| KIAA1012 | A_23_P107552 | 0.475881791 | 0.025177431 |
| BDH2 | A_23_P92490 | 0.475946441 | 0.025154848 |
| LIN9 | A_32_P233304 | 0.476489416 | 0.024965823 |
| DDX52 | A_23_P118660 | 0.4767525 | 0.024874645 |
| ZNF841 | A_23_P27404 | 0.476780923 | 0.02486481 |
| RPS10 | A_24_P418619 | 0.476794555 | 0.024860095 |
| HSPE1 | A_23_P56922 | 0.476808997 | 0.0248551 |
| XRCC1 | A_23_P153692 | 0.477204831 | 0.0247185 |
| ZNF791 | A_24_P382401 | 0.477260422 | 0.024699364 |
| ZNF8 | A_23_P130470 | 0.477354057 | 0.024667159 |
| CYP20A1 | A_32_P110433 | 0.477446783 | 0.0246353 |
| CCNB2 | A_23_P65757 | 0.477485839 | 0.024621891 |
| MAP3K7IP2 | A_23_P19702 | 0.477933409 | 0.024468641 |
| METT5D1 | A_32_P183495 | 0.478163528 | 0.024390145 |
| ELK4 | A_24_P902091 | 0.478269196 | 0.024354167 |
| ATP13A1 | A_23_P5163 | 0.478405956 | 0.024307667 |
| ZNF671 | A_23_P50217 | 0.478478641 | 0.024282982 |
| HOXA3 | A_23_P501538 | 0.478548341 | 0.024259329 |
| ZFX | A_24_P940524 | 0.478817164 | 0.024168278 |
| ARSG | A_24_P339416 | 0.478916084 | 0.024134842 |
| LOC100291647 | A_32_P82383 | 0.479128611 | 0.02406313 |
| ZNF443 | A_23_P67278 | 0.479208455 | 0.024036233 |
| RNPEP | A_23_P97770 | 0.479242744 | 0.02402469 |
| FMN2 | A_32_P229132 | 0.4793151 | 0.024000345 |
| FOXL2 | A_23_P110052 | 0.479406089 | 0.02396976 |
| CDO1 | A_23_P30294 | 0.479406598 | 0.023969588 |
| C1orf56 | A_24_P94351 | 0.479622231 | 0.023897229 |
| LSM2 | A_23_P59153 | 0.479639314 | 0.023891504 |
| LOC158381 | A_24_P358245 | 0.479719669 | 0.023864589 |
| RPS10 | A_32_P119204 | 0.479719788 | 0.02386455 |
| GZF1 | A_24_P156288 | 0.479725411 | 0.023862667 |
| ORAI3 | A_23_P106898 | 0.480122839 | 0.023729915 |
| KLHL36 | A_24_P184937 | 0.480284913 | 0.023675947 |
| TATDN1 | A_23_P254978 | 0.480467128 | 0.02361539 |
| CHD8 | A_24_P361167 | 0.480549345 | 0.023588107 |
| CROCCL2 | A_32_P384562 | 0.480799342 | 0.023505302 |
| DYX1C1 | A_23_P37505 | 0.480981711 | 0.023445043 |
| C20orf4 | A_23_P166196 | 0.481221916 | 0.023365863 |
| TAF2 | A_24_P16892 | 0.481230585 | 0.02336301 |
| ZNF350 | A_23_P78458 | 0.481235934 | 0.023361249 |
| POU4F1 | A_23_P205164 | 0.481298705 | 0.023340596 |
| C15orf44 | A_23_P37475 | 0.482015384 | 0.023105829 |
| ZNF177 | A_24_P168398 | 0.482201723 | 0.023045099 |
| NID1 | A_23_P200928 | 0.482399512 | 0.022980778 |
| NR3C1 | A_23_P214059 | 0.482488098 | 0.022952016 |
| CHRNB1 | A_23_P207106 | 0.482504239 | 0.022946778 |
| TPR | A_24_P179611 | 0.482539065 | 0.022935481 |
| WDR90 | A_23_P373992 | 0.482808099 | 0.022848359 |
| MUTED | A_24_P77870 | 0.482957515 | 0.022800088 |
| SYNRG | A_23_P207541 | 0.483128927 | 0.022744811 |
| SUV39H1 | A_23_P422193 | 0.48318485 | 0.022726801 |
| C6orf204 | A_32_P49832 | 0.483411922 | 0.022653786 |
| ZNF318 | A_23_P145175 | 0.483475938 | 0.022633236 |
| ZNF285A | A_23_P101319 | 0.483599624 | 0.022593573 |
| LOC400756 | A_32_P887687 | 0.48400089 | 0.022465281 |
| YBX2 | A_23_P49865 | 0.484022006 | 0.022458546 |
| RBBP9 | A_23_P257538 | 0.484068103 | 0.022443849 |
| PGM3 | A_23_P19592 | 0.484079776 | 0.022440129 |
| ZCCHC10 | A_23_P7577 | 0.484149527 | 0.022417907 |
| METTL7A | A_24_P941773 | 0.484201137 | 0.022401477 |
| C2orf76 | A_23_P39542 | 0.484434112 | 0.022327428 |
| LYSMD1 | A_23_P430381 | 0.484473071 | 0.022315064 |
| DUSP12 | A_23_P51508 | 0.484673461 | 0.022251557 |
| ZNF70 | A_24_P941625 | 0.484932325 | 0.022169733 |
| RAE1 | A_23_P346206 | 0.485024256 | 0.022140733 |
| SHPRH | A_24_P153576 | 0.485047707 | 0.02213334 |
| CD99 | A_24_P70993 | 0.485098416 | 0.022117361 |
| TADA2A | A_23_P66664 | 0.485209353 | 0.022082435 |
| ZNF395 | A_23_P157460 | 0.485283927 | 0.022058982 |
| CCDC146 | A_23_P168771 | 0.485394054 | 0.022024385 |
| PDIA5 | A_23_P167040 | 0.485547525 | 0.021976243 |
| SPAG1 | A_23_P146066 | 0.486125952 | 0.021795556 |
| LCMT2 | A_23_P106505 | 0.48614223 | 0.021790489 |
| C1orf77 | A_24_P265539 | 0.486683705 | 0.021622458 |
| MECOM | A_24_P15154 | 0.486789006 | 0.021589902 |
| PLK1 | A_24_P313504 | 0.486813258 | 0.021582409 |
| CCDC88C | A_32_P170811 | 0.486845783 | 0.021572364 |
| ESCO2 | A_24_P323598 | 0.487178757 | 0.021469744 |
| PCBD2 | A_24_P200603 | 0.487420498 | 0.021395487 |
| CCDC142 | A_23_P424712 | 0.487627857 | 0.021331956 |
| ZNF557 | A_32_P177097 | 0.487734802 | 0.021299249 |
| HP | A_23_P206760 | 0.487952987 | 0.021232646 |
| YTHDF1 | A_24_P36425 | 0.487971059 | 0.021227137 |
| ZMYM1 | A_23_P161091 | 0.48808936 | 0.021191102 |
| CASC1 | A_23_P95231 | 0.488154026 | 0.021171425 |
| NEIL3 | A_23_P155711 | 0.4883789 | 0.021103115 |
| FIZ1 | A_23_P107855 | 0.488439143 | 0.021084845 |
| RPS10 | A_32_P86728 | 0.488442986 | 0.02108368 |
| GORASP2 | A_24_P328320 | 0.489070326 | 0.020894186 |
| TTRAP | A_23_P8311 | 0.489197454 | 0.020855953 |
| CNIH4 | A_23_P200507 | 0.490046466 | 0.020602057 |
| RAGE | A_23_P76731 | 0.490336881 | 0.020515781 |
| RNF111 | A_23_P77160 | 0.490540433 | 0.020455483 |
| URB2 | A_23_P74914 | 0.490616151 | 0.02043309 |
| RPP38 | A_23_P150080 | 0.490617194 | 0.020432782 |
| KRTCAP3 | A_23_P433132 | 0.490636739 | 0.020427005 |
| RC3H1 | A_32_P96807 | 0.490750883 | 0.020393292 |
| C6orf97 | A_23_P93514 | 0.490949243 | 0.020334813 |
| ZMYM6 | A_24_P25326 | 0.490986458 | 0.020323857 |
| LOC100132733 | A_32_P109604 | 0.491277052 | 0.020238467 |
| TP53RK | A_23_P109055 | 0.491487916 | 0.020176686 |
| PLA2G4A | A_23_P11685 | 0.491637774 | 0.020132871 |
| SMARCA5 | A_23_P256716 | 0.491694569 | 0.020116286 |
| FAM96A | A_23_P37636 | 0.491929883 | 0.020047686 |
| BLZF1 | A_23_P23266 | 0.4919489 | 0.020042151 |
| ZNF132 | A_24_P121846 | 0.492100148 | 0.019998168 |
| ERO1LB | A_23_P97218 | 0.492266152 | 0.019949983 |
| FAM175A | A_23_P253464 | 0.492418029 | 0.01990598 |
| CXXC1 | A_23_P141779 | 0.492589555 | 0.019856379 |
| ANTXR2 | A_23_P170733 | 0.492801261 | 0.019795296 |
| C21orf49 | A_24_P359322 | 0.492957203 | 0.019750399 |
| MFAP1 | A_23_P14636 | 0.493076449 | 0.019716122 |
| SEC24B | A_23_P69683 | 0.493230012 | 0.019672052 |
| C1orf88 | A_32_P215700 | 0.493311562 | 0.019648681 |
| EPB41L4A | A_24_P944378 | 0.493447806 | 0.019609685 |
| CKS1B | A_32_P192430 | 0.493637334 | 0.019555541 |
| PSMB3 | A_23_P100576 | 0.493766642 | 0.01951867 |
| SNX27 | A_23_P201238 | 0.493795729 | 0.019510384 |
| HOXC13 | A_23_P64808 | 0.493945995 | 0.019467622 |
| C1orf35 | A_23_P97328 | 0.493948626 | 0.019466874 |
| FAM122C | A_23_P340188 | 0.494012565 | 0.019448701 |
| ESCO1 | A_32_P223319 | 0.494043954 | 0.019439785 |
| DYNC2LI1 | A_23_P502174 | 0.494115996 | 0.019419334 |
| TPR | A_23_P45875 | 0.494160562 | 0.019406692 |
| C14orf142 | A_23_P99579 | 0.494172416 | 0.01940333 |
| GATA2 | A_24_P165998 | 0.494298794 | 0.01936752 |
| METTL5 | A_23_P142634 | 0.495084034 | 0.019146207 |
| RIN2 | A_24_P305570 | 0.49527282 | 0.019093305 |
| ZNF564 | A_23_P55880 | 0.495325925 | 0.019078445 |
| LGMN | A_23_P25994 | 0.495678427 | 0.018980042 |
| CSTF1 | A_23_P362824 | 0.495817 | 0.018941471 |
| MAMDC4 | A_23_P71790 | 0.496098397 | 0.018863339 |
| DTWD1 | A_23_P3212 | 0.496287872 | 0.018810877 |
| TEKT1 | A_23_P15678 | 0.496370105 | 0.018788145 |
| LILRB5 | A_23_P4773 | 0.496462663 | 0.018762585 |
| ZNF616 | A_32_P186921 | 0.496564771 | 0.018734421 |
| KIAA1009 | A_24_P337546 | 0.496659927 | 0.018708204 |
| CRTC2 | A_23_P12178 | 0.496763485 | 0.018679707 |
| RANGRF | A_23_P254498 | 0.496909663 | 0.018639541 |
| SMC1A | A_23_P217411 | 0.497231843 | 0.018551259 |
| SSR1 | A_24_P544882 | 0.497258388 | 0.018544 |
| HIBCH | A_23_P154349 | 0.497319219 | 0.018527375 |
| HES1 | A_24_P938293 | 0.497615977 | 0.018446442 |
| SYCP2L | A_23_P347777 | 0.49765076 | 0.018436975 |
| LOC644246 | A_24_P221327 | 0.497821922 | 0.018390444 |
| DIDO1 | A_23_P388190 | 0.49782777 | 0.018388856 |
| CDC42BPG | A_23_P64051 | 0.497872928 | 0.018376596 |
| PCDH10 | A_23_P158851 | 0.497898786 | 0.018369579 |
| SDR39U1 | A_23_P163161 | 0.497991659 | 0.018344394 |
| FAM165B | A_23_P82051 | 0.498295604 | 0.018262166 |
| ZNF213 | A_24_P364845 | 0.498351107 | 0.018247183 |
| C2orf49 | A_24_P883629 | 0.498720782 | 0.01814764 |
| DCTN6 | A_23_P43049 | 0.498861007 | 0.018109996 |
| EFNA4 | A_23_P322 | 0.499081384 | 0.018050962 |
| YIPF3 | A_23_P111005 | 0.499090367 | 0.018048559 |
| LYRM2 | A_23_P42042 | 0.499330741 | 0.017984353 |
| SDCCAG8 | A_24_P179489 | 0.499485431 | 0.017943131 |
| SPA17 | A_23_P104876 | 0.499486123 | 0.017942947 |
| DMPK | A_23_P50535 | 0.499896698 | 0.017833909 |
| ERGIC3 | A_24_P216308 | 0.500004932 | 0.017805254 |
| MED1 | A_23_P425704 | 0.500022981 | 0.017800479 |
| PIAS3 | A_23_P149678 | 0.500058716 | 0.017791028 |
| ZNF180 | A_23_P89921 | 0.500110158 | 0.017777431 |
| ZNF551 | A_24_P68019 | 0.500422382 | 0.017695082 |
| GJA1 | A_23_P93591 | 0.500464349 | 0.017684037 |
| ZNF547 | A_23_P333592 | 0.500666808 | 0.01763083 |
| ZNF224 | A_23_P10885 | 0.500872052 | 0.017577023 |
| RPS24 | A_23_P1206 | 0.501066379 | 0.017526201 |
| HPR | A_23_P421493 | 0.501068386 | 0.017525677 |
| GON4L | A_24_P276853 | 0.501558033 | 0.017398149 |
| SEC63 | A_23_P214977 | 0.501653919 | 0.017373263 |
| TRMT112 | A_23_P12992 | 0.501708138 | 0.017359205 |
| SNX14 | A_23_P259054 | 0.501746329 | 0.017349308 |
| GAS1 | A_23_P83134 | 0.50205488 | 0.017269514 |
| ZNF791 | A_23_P50418 | 0.502091945 | 0.017259948 |
| WDR16 | A_23_P4074 | 0.502237246 | 0.017222492 |
| XPO4 | A_24_P333716 | 0.502534814 | 0.017145988 |
| THAP2 | A_23_P105382 | 0.502681757 | 0.017108311 |
| CBX8 | A_23_P55421 | 0.502932814 | 0.017044094 |
| CETN3 | A_23_P7732 | 0.503024247 | 0.017020755 |
| SLC38A3 | A_24_P402784 | 0.503452479 | 0.016911789 |
| MANEA | A_23_P255663 | 0.504466074 | 0.016656121 |
| SDHC | A_24_P233850 | 0.504589625 | 0.016625171 |
| CDKAL1 | A_23_P44781 | 0.505191939 | 0.016474959 |
| GLIS3 | A_23_P384023 | 0.505211503 | 0.016470098 |
| SPEF1 | A_24_P211420 | 0.505230226 | 0.016465448 |
| ABCB10 | A_23_P201918 | 0.505382047 | 0.016427776 |
| AURKA | A_23_P131866 | 0.505468851 | 0.016406269 |
| RAB5C | A_23_P107211 | 0.505514551 | 0.016394955 |
| LOC100287878 | A_23_P46660 | 0.505856114 | 0.016310594 |
| ZNF426 | A_23_P101351 | 0.5061623 | 0.016235271 |
| C10orf12 | A_23_P115838 | 0.506725169 | 0.016097536 |
| UNC50 | A_23_P252700 | 0.507205806 | 0.015980675 |
| WAC | A_23_P201998 | 0.507251916 | 0.0159695 |
| SYCP2 | A_24_P333644 | 0.507333648 | 0.015949707 |
| CTDSPL2 | A_32_P76060 | 0.507662309 | 0.015870318 |
| CLK2 | A_24_P67027 | 0.507945281 | 0.015802222 |
| CCNB1 | A_23_P122197 | 0.508127105 | 0.015758592 |
| RBM16 | A_23_P111303 | 0.50819866 | 0.015741449 |
| ADAR | A_23_P200439 | 0.508921531 | 0.015569109 |
| KIAA0494 | A_23_P11564 | 0.508961169 | 0.015559703 |
| PAQR3 | A_23_P337875 | 0.509134907 | 0.015518531 |
| SEMA4G | A_23_P127068 | 0.509162307 | 0.015512046 |
| RNF138 | A_23_P89755 | 0.509514384 | 0.015428911 |
| CRNKL1 | A_23_P131967 | 0.510192206 | 0.015269876 |
| JUB | A_23_P54055 | 0.510514371 | 0.015194755 |
| CENPA | A_24_P413884 | 0.510764253 | 0.015136695 |
| PIBF1 | A_23_P411881 | 0.510867491 | 0.015112761 |
| NOL8 | A_24_P360601 | 0.510915228 | 0.015101704 |
| NUP153 | A_23_P122254 | 0.510937376 | 0.015096576 |
| PDE3A | A_23_P60837 | 0.510976653 | 0.015087487 |
| ANKRD26 | A_23_P115597 | 0.511065497 | 0.015066942 |
| DUSP5P | A_32_P157846 | 0.511450171 | 0.014978249 |
| FAM54A | A_23_P253752 | 0.511743114 | 0.014910991 |
| FBXO46 | A_23_P27744 | 0.512370697 | 0.014767727 |
| WDR92 | A_32_P165112 | 0.512565761 | 0.014723427 |
| CCDC111 | A_23_P358470 | 0.512662915 | 0.014701403 |
| CD28 | A_23_P91095 | 0.513041433 | 0.014615851 |
| NIN | A_24_P928361 | 0.513064702 | 0.014610605 |
| DCP1B | A_23_P313512 | 0.513211699 | 0.0145775 |
| TEX14 | A_23_P54996 | 0.51328976 | 0.014559944 |
| FAM183B | A_24_P391991 | 0.513990687 | 0.01440308 |
| FBXO4 | A_24_P171873 | 0.514023342 | 0.014395805 |
| MRPS14 | A_23_P62731 | 0.514062101 | 0.014387175 |
| MOBKL3 | A_23_P210274 | 0.514064201 | 0.014386707 |
| CDCA2 | A_24_P323434 | 0.514337315 | 0.014326015 |
| SPG11 | A_23_P65699 | 0.51461285 | 0.014264995 |
| TIGD4 | A_23_P370684 | 0.515317621 | 0.014109879 |
| NETO2 | A_23_P3681 | 0.515415438 | 0.014088459 |
| MLF1 | A_24_P345679 | 0.515791988 | 0.014006248 |
| HOXB2 | A_23_P107283 | 0.51588127 | 0.013986813 |
| LRTOMT | A_23_P98763 | 0.515942276 | 0.013973545 |
| PRKD3 | A_24_P165656 | 0.51669541 | 0.013810597 |
| ZNF823 | A_23_P39050 | 0.517014697 | 0.013741984 |
| QRSL1 | A_23_P82181 | 0.517065156 | 0.013731167 |
| C9orf9 | A_23_P32135 | 0.517413025 | 0.013656776 |
| TDH | A_24_P334845 | 0.517557844 | 0.013625903 |
| CENPF | A_24_P96780 | 0.517665 | 0.013603097 |
| ZNF521 | A_23_P159027 | 0.517910903 | 0.013550877 |
| PLD2 | A_23_P4308 | 0.517966685 | 0.013539054 |
| GON4L | A_23_P97250 | 0.518088659 | 0.01351323 |
| TROVE2 | A_24_P222684 | 0.518323029 | 0.013463724 |
| CTNND1 | A_24_P881527 | 0.518585985 | 0.013408355 |
| C15orf42 | A_23_P345707 | 0.518795397 | 0.013364394 |
| KAL1 | A_23_P429950 | 0.518833278 | 0.013356454 |
| BTBD7 | A_24_P230808 | 0.518845777 | 0.013353835 |
| DNAI2 | A_23_P169966 | 0.518877327 | 0.013347226 |
| LIG1 | A_23_P39116 | 0.519002249 | 0.013321085 |
| RAD51AP1 | A_23_P99292 | 0.519037675 | 0.01331368 |
| TMEM188 | A_23_P397417 | 0.51934016 | 0.013250582 |
| POLD3 | A_24_P75056 | 0.51940745 | 0.013236579 |
| KCNJ5 | A_23_P202927 | 0.519595886 | 0.013197429 |
| MLF1 | A_23_P143906 | 0.519633574 | 0.013189611 |
| C20orf72 | A_23_P102769 | 0.519743168 | 0.013166895 |
| DIDO1 | A_24_P322611 | 0.519909714 | 0.013132437 |
| ADAMTSL4 | A_23_P115011 | 0.520154591 | 0.013081906 |
| TOR1AIP1 | A_23_P161074 | 0.520444592 | 0.013022268 |
| TTC29 | A_23_P92517 | 0.520484859 | 0.013014005 |
| DDX59 | A_24_P156886 | 0.520536218 | 0.013003472 |
| MORN5 | A_24_P401491 | 0.520602626 | 0.012989862 |
| ZBTB39 | A_23_P13683 | 0.520994034 | 0.012909885 |
| ZNFX1 | A_23_P68462 | 0.52117097 | 0.012873863 |
| LMNB1 | A_23_P258493 | 0.521355274 | 0.012836428 |
| TGFBR3 | A_23_P200780 | 0.521516924 | 0.012803668 |
| AKR1A1 | A_24_P79750 | 0.521780033 | 0.012750492 |
| KIF27 | A_24_P109071 | 0.521938417 | 0.012718568 |
| PAX9 | A_32_P70818 | 0.521943716 | 0.012717501 |
| OIP5 | A_23_P379614 | 0.521998459 | 0.012706483 |
| SCAND2 | A_23_P205875 | 0.522118792 | 0.012682291 |
| UBL5 | A_23_P78563 | 0.522691962 | 0.012567576 |
| PLEKHA5 | A_23_P218068 | 0.523019838 | 0.012502336 |
| NME7 | A_23_P46063 | 0.523059956 | 0.012494373 |
| CCDC40 | A_24_P160680 | 0.52324556 | 0.012457583 |
| KIAA1407 | A_23_P419213 | 0.523736283 | 0.012360741 |
| C13orf23 | A_23_P205101 | 0.523860299 | 0.012336365 |
| REV1 | A_24_P291426 | 0.52415694 | 0.012278216 |
| RBPMS2 | A_23_P100056 | 0.524236506 | 0.012262657 |
| C6orf167 | A_32_P95914 | 0.524310133 | 0.012248274 |
| ABCC9 | A_23_P204194 | 0.524326085 | 0.01224516 |
| IQCH | A_23_P77212 | 0.524817179 | 0.012149597 |
| ABCB10 | A_32_P23624 | 0.525123346 | 0.012090328 |
| ZFP161 | A_24_P74064 | 0.525358522 | 0.012044962 |
| C6orf170 | A_23_P326325 | 0.525856506 | 0.01194936 |
| ATN1 | A_23_P13885 | 0.525866601 | 0.011947428 |
| MAGOHB | A_24_P828949 | 0.525937901 | 0.011933793 |
| MCM8 | A_23_P68547 | 0.526131083 | 0.011896912 |
| ARHGAP19 | A_23_P1387 | 0.526264132 | 0.011871566 |
| ZNF79 | A_23_P303763 | 0.526572168 | 0.011813054 |
| ZNF223 | A_24_P99838 | 0.526660939 | 0.011796235 |
| CCDC138 | A_23_P311144 | 0.526832529 | 0.011763781 |
| SNAP29 | A_24_P48862 | 0.526954306 | 0.011740793 |
| LRIT2 | A_24_P478350 | 0.526993019 | 0.011733493 |
| PEX11A | A_23_P37560 | 0.527228099 | 0.011689243 |
| TGIF1 | A_23_P153197 | 0.527244949 | 0.011686076 |
| BCKDHB | A_23_P93464 | 0.527251457 | 0.011684853 |
| ANKHD1 | A_23_P213326 | 0.527502236 | 0.011637813 |
| PIGM | A_23_P103248 | 0.527624379 | 0.011614958 |
| RNFT1 | A_23_P207299 | 0.527931238 | 0.011557701 |
| TTC13 | A_23_P103864 | 0.52839523 | 0.011471564 |
| RNF5 | A_23_P320200 | 0.528503806 | 0.011451483 |
| KIAA1958 | A_23_P412764 | 0.528790878 | 0.011398529 |
| AATF | A_23_P89460 | 0.528800831 | 0.011396697 |
| WDR90 | A_23_P411833 | 0.529069803 | 0.01134727 |
| KIF4A | A_23_P148475 | 0.529245222 | 0.011315129 |
| INHBB | A_23_P153964 | 0.530092337 | 0.011160965 |
| NCBP1 | A_23_P257104 | 0.530843091 | 0.011025778 |
| C10orf119 | A_23_P202594 | 0.530922829 | 0.011011499 |
| NUP43 | A_24_P200761 | 0.531160999 | 0.010968938 |
| DCXR | A_23_P44166 | 0.531172753 | 0.010966842 |
| CARD8 | A_23_P27721 | 0.531207622 | 0.010960623 |
| C16orf53 | A_23_P77422 | 0.5312738 | 0.010948828 |
| SNRPE | A_23_P126291 | 0.531345444 | 0.010936071 |
| ZNF549 | A_23_P399146 | 0.531540436 | 0.010901412 |
| C5orf54 | A_23_P259103 | 0.531552707 | 0.010899234 |
| PARP1 | A_23_P114783 | 0.531668118 | 0.010878766 |
| CCDC12 | A_23_P372334 | 0.531878161 | 0.010841596 |
| PBX1 | A_23_P62953 | 0.532256126 | 0.010774972 |
| ZNF232 | A_23_P4294 | 0.532281854 | 0.010770449 |
| SCAND2 | A_24_P90792 | 0.532411765 | 0.010747635 |
| WDR92 | A_32_P165113 | 0.532675285 | 0.010701479 |
| CKS1B | A_23_P45917 | 0.533002893 | 0.010644324 |
| CECR4 | A_32_P131143 | 0.533042287 | 0.010637468 |
| LOC100132240 | A_32_P63562 | 0.533064891 | 0.010633536 |
| VPS37B | A_23_P150876 | 0.533146543 | 0.010619342 |
| ITGA6 | A_23_P210176 | 0.533349871 | 0.010584063 |
| SCNM1 | A_23_P34968 | 0.533664529 | 0.010529658 |
| SMEK2 | A_24_P194931 | 0.534149481 | 0.010446256 |
| C14orf45 | A_23_P76983 | 0.534289602 | 0.01042226 |
| PCM1 | A_24_P135167 | 0.534314026 | 0.010418081 |
| KRTCAP2 | A_23_P149249 | 0.535057143 | 0.010291614 |
| ARNT | A_24_P391568 | 0.535171724 | 0.010272226 |
| NUP54 | A_23_P92320 | 0.535229874 | 0.010262398 |
| KIAA0907 | A_23_P74467 | 0.535260032 | 0.010257304 |
| HOXA4 | A_23_P253982 | 0.535496267 | 0.010217475 |
| MIA3 | A_24_P255516 | 0.536205375 | 0.010098679 |
| SDHD | A_32_P60185 | 0.536217319 | 0.010096687 |
| C1orf27 | A_23_P284 | 0.536771775 | 0.010004607 |
| ZNF624 | A_23_P153037 | 0.536870753 | 0.009988242 |
| RFWD2 | A_23_P92292 | 0.536913479 | 0.009981184 |
| SNAPC1 | A_23_P37244 | 0.537011794 | 0.00996496 |
| ZNF222 | A_24_P165082 | 0.538445629 | 0.009730794 |
| CENPF | A_23_P401 | 0.538643061 | 0.009698908 |
| MTA2 | A_23_P203420 | 0.538712242 | 0.009687755 |
| COQ2 | A_23_P124733 | 0.53895366 | 0.009648918 |
| USP37 | A_24_P15114 | 0.539018153 | 0.009638565 |
| EFHC1 | A_32_P86245 | 0.539636013 | 0.009539839 |
| HSCB | A_23_P40588 | 0.539670626 | 0.009534333 |
| CDC14B | A_23_P216679 | 0.540079641 | 0.009469466 |
| PIGC | A_23_P86252 | 0.540318198 | 0.009431801 |
| TRDMT1 | A_23_P115636 | 0.540529599 | 0.009398526 |
| PATL1 | A_23_P373079 | 0.540557981 | 0.009394066 |
| TMEM67 | A_23_P429581 | 0.540564027 | 0.009393116 |
| SPAG8 | A_24_P412036 | 0.540938233 | 0.00933448 |
| LTBP1 | A_23_P43810 | 0.541264624 | 0.009283582 |
| PPIL6 | A_24_P925389 | 0.541747668 | 0.009208675 |
| LOC100289794 | A_32_P37461 | 0.542385793 | 0.009110481 |
| MAPKSP1 | A_23_P110362 | 0.542666786 | 0.009067517 |
| WIPF2 | A_24_P838748 | 0.542950935 | 0.009024239 |
| ILF2 | A_23_P257956 | 0.543093451 | 0.009002597 |
| GEMIN8 | A_24_P156576 | 0.543318244 | 0.008968547 |
| TAF1L | A_23_P419850 | 0.543547199 | 0.008933976 |
| ROBLD3 | A_24_P182433 | 0.543652596 | 0.008918099 |
| FOXA1 | A_23_P37127 | 0.544046501 | 0.008858965 |
| METTL9 | A_24_P112984 | 0.54451712 | 0.008788738 |
| LPIN1 | A_32_P52609 | 0.545045913 | 0.008710377 |
| ZNF274 | A_23_P153251 | 0.545484828 | 0.008645773 |
| DPM1 | A_23_P68472 | 0.545718669 | 0.008611515 |
| MYST3 | A_23_P407628 | 0.545985208 | 0.008572604 |
| TMEM223 | A_23_P1956 | 0.546043748 | 0.008564077 |
| C8orf42 | A_23_P393686 | 0.546307655 | 0.008525724 |
| TMEM131 | A_23_P368558 | 0.546592141 | 0.008484539 |
| SCNM1 | A_24_P259799 | 0.546779218 | 0.008457545 |
| CHIC2 | A_23_P110345 | 0.546940892 | 0.008434274 |
| DEK | A_23_P254702 | 0.54710029 | 0.008411382 |
| SNX5 | A_23_P131954 | 0.547168425 | 0.008401613 |
| KIFC1 | A_23_P133956 | 0.547323599 | 0.008379398 |
| ATP6V0D2 | A_23_P146146 | 0.547564113 | 0.008345061 |
| ADNP | A_23_P254179 | 0.548178223 | 0.008257915 |
| GSR | A_32_P31618 | 0.550479059 | 0.007938055 |
| BMS1 | A_32_P11450 | 0.550493235 | 0.007936116 |
| ATHL1 | A_23_P98686 | 0.550779434 | 0.007897062 |
| RIT1 | A_23_P115052 | 0.552332693 | 0.007687863 |
| HES1 | A_23_P6596 | 0.552419168 | 0.007676352 |
| MITD1 | A_23_P311020 | 0.552662678 | 0.007644014 |
| CRYBB1 | A_23_P143621 | 0.55272542 | 0.0076357 |
| ZNF234 | A_23_P153286 | 0.552771215 | 0.007629636 |
| TBX5 | A_24_P30557 | 0.552829501 | 0.007621925 |
| NUP133 | A_23_P12503 | 0.552850349 | 0.007619168 |
| C17orf80 | A_23_P207927 | 0.552891914 | 0.007613674 |
| METTL14 | A_23_P110243 | 0.552958013 | 0.007604944 |
| NPHP1 | A_24_P88801 | 0.553459877 | 0.007538932 |
| CTSW | A_24_P396167 | 0.553966734 | 0.007472744 |
| ALG10 | A_23_P204333 | 0.554045481 | 0.007462504 |
| FLJ27352 | A_32_P447001 | 0.55441823 | 0.007414191 |
| HOTAIR | A_32_P168442 | 0.554714824 | 0.007375933 |
| hCG_17324 | A_32_P67526 | 0.555323184 | 0.007297971 |
| GSTM2 | A_23_P397208 | 0.556702754 | 0.007123698 |
| CCDC135 | A_23_P129367 | 0.556871724 | 0.007102592 |
| IKBKAP | A_23_P169189 | 0.556940832 | 0.007093974 |
| UBA6 | A_24_P166144 | 0.557795164 | 0.006988155 |
| GRHL2 | A_24_P35228 | 0.557875299 | 0.006978297 |
| HIST1H3A | A_23_P111037 | 0.557884764 | 0.006977133 |
| PEX7 | A_23_P93543 | 0.557945847 | 0.006969628 |
| POLR3C | A_23_P35256 | 0.558192364 | 0.006939404 |
| HERC5 | A_23_P110196 | 0.558686424 | 0.006879158 |
| LOC100132351 | A_32_P146579 | 0.55872187 | 0.006874852 |
| KIAA0586 | A_23_P99710 | 0.558985222 | 0.006842932 |
| RSPH4A | A_32_P137920 | 0.559165438 | 0.00682116 |
| HOXA9 | A_23_P500998 | 0.559455119 | 0.006786283 |
| BAT2D1 | A_23_P412409 | 0.559722658 | 0.006754203 |
| SLC16A6 | A_24_P731648 | 0.559931995 | 0.00672919 |
| POU3F2 | A_23_P82026 | 0.56017437 | 0.006700325 |
| ZNF432 | A_24_P65658 | 0.560592297 | 0.006650794 |
| CHCHD1 | A_23_P371613 | 0.561079247 | 0.006593465 |
| MCM8 | A_24_P305556 | 0.56153898 | 0.006539717 |
| LYRM2 | A_23_P42036 | 0.561822822 | 0.006506714 |
| C3orf15 | A_32_P55871 | 0.562198323 | 0.006463266 |
| ZNF408 | A_23_P32722 | 0.562362662 | 0.006444327 |
| C2orf29 | A_23_P108761 | 0.562690348 | 0.0064067 |
| PLSCR4 | A_23_P91910 | 0.564807614 | 0.006167947 |
| ZNF222 | A_23_P218517 | 0.564921558 | 0.00615531 |
| ZNF564 | A_24_P14328 | 0.565257665 | 0.00611816 |
| POU3F4 | A_23_P114210 | 0.565538243 | 0.006087291 |
| DUSP18 | A_24_P83118 | 0.565595534 | 0.006081003 |
| SPATA24 | A_32_P53852 | 0.565643909 | 0.006075699 |
| MRPL9 | A_23_P34956 | 0.565796882 | 0.00605895 |
| ZNF226 | A_23_P395555 | 0.565940409 | 0.006043269 |
| LOC100287126 | A_32_P188 | 0.565950501 | 0.006042168 |
| C5orf39 | A_23_P431591 | 0.566132573 | 0.006022328 |
| TSEN15 | A_23_P104022 | 0.566399813 | 0.005993306 |
| LCA5 | A_23_P418083 | 0.567047379 | 0.005923462 |
| DDB1 | A_23_P258246 | 0.567690661 | 0.00585475 |
| METTL13 | A_23_P201672 | 0.56915266 | 0.005701046 |
| MTR | A_24_P67806 | 0.569579466 | 0.005656813 |
| LIPT1 | A_23_P501805 | 0.570129785 | 0.0056002 |
| C8orf12 | A_23_P256965 | 0.570255883 | 0.005587294 |
| SNAPIN | A_23_P161022 | 0.570327616 | 0.005579964 |
| USP37 | A_24_P109082 | 0.570466764 | 0.005565767 |
| NUP43 | A_23_P31055 | 0.570955513 | 0.005516138 |
| SLC40A1 | A_23_P102391 | 0.571162988 | 0.005495183 |
| TSEN15 | A_23_P104025 | 0.572728682 | 0.005339166 |
| GSTM1 | A_23_P115407 | 0.573291851 | 0.005283957 |
| SMC2 | A_23_P60271 | 0.573622554 | 0.00525176 |
| OTOA | A_32_P52519 | 0.573854147 | 0.005229309 |
| SEC23B | A_23_P6119 | 0.573965021 | 0.005218589 |
| ZNF473 | A_24_P941376 | 0.57418828 | 0.005197058 |
| KPNA5 | A_23_P156443 | 0.574189953 | 0.005196897 |
| ARMC2 | A_23_P254140 | 0.574346611 | 0.005181834 |
| CDCA2 | A_23_P385861 | 0.574492406 | 0.005167848 |
| H2AFV | A_23_P145904 | 0.574871439 | 0.005131635 |
| C5orf54 | A_24_P166311 | 0.57492739 | 0.005126307 |
| FOXJ1 | A_23_P348636 | 0.575853925 | 0.00503875 |
| NUF2 | A_23_P74349 | 0.575860631 | 0.005038121 |
| UBXN2A | A_23_P131240 | 0.576300907 | 0.004996958 |
| LOC100294362 | A_23_P372074 | 0.576755021 | 0.004954794 |
| PRPSAP1 | A_23_P15305 | 0.576987489 | 0.004933326 |
| PLEKHA7 | A_24_P348806 | 0.577118325 | 0.004921277 |
| ALMS1 | A_23_P312174 | 0.577323978 | 0.004902387 |
| SASS6 | A_24_P349151 | 0.578062432 | 0.004835057 |
| IRAK1BP1 | A_32_P21474 | 0.578953009 | 0.004754884 |
| ZNF256 | A_23_P16022 | 0.579015197 | 0.004749327 |
| KIF14 | A_23_P149668 | 0.579110024 | 0.004740864 |
| CCDC49 | A_23_P38115 | 0.579896333 | 0.004671174 |
| ZNF226 | A_24_P930347 | 0.580438406 | 0.004623629 |
| SDHD | A_32_P155631 | 0.580478576 | 0.004620122 |
| OGFOD1 | A_23_P3775 | 0.580585946 | 0.004610759 |
| TOR1AIP1 | A_24_P267997 | 0.580631179 | 0.004606819 |
| C1orf192 | A_32_P331139 | 0.580670504 | 0.004603396 |
| LSM5 | A_23_P93750 | 0.580755994 | 0.004595962 |
| HIBCH | A_23_P154345 | 0.581548043 | 0.004527563 |
| LOC399959 | A_24_P794447 | 0.58296554 | 0.004407271 |
| USP21 | A_23_P126689 | 0.583204653 | 0.004387245 |
| HERC4 | A_24_P346807 | 0.583421724 | 0.004369131 |
| LOC100133315 | A_32_P437876 | 0.583446238 | 0.004367089 |
| HNRNPL | A_23_P119677 | 0.583534137 | 0.004359774 |
| LRRC39 | A_23_P330578 | 0.58394259 | 0.00432592 |
| CDT1 | A_23_P37704 | 0.584313104 | 0.0042954 |
| BOLA1 | A_23_P46507 | 0.584515091 | 0.004278838 |
| CHMP2B | A_23_P212535 | 0.584532288 | 0.00427743 |
| ZNF613 | A_24_P173234 | 0.584547837 | 0.004276158 |
| ZBTB41 | A_32_P108826 | 0.584694495 | 0.004264172 |
| ZNF20 | A_23_P16242 | 0.586303813 | 0.004134483 |
| TGIF2 | A_23_P79794 | 0.586929749 | 0.00408494 |
| ZNF222 | A_23_P125042 | 0.589342406 | 0.003898594 |
| CDC73 | A_23_P137731 | 0.590270568 | 0.003828827 |
| C1orf156 | A_23_P85726 | 0.590593158 | 0.003804825 |
| ZNF136 | A_23_P67312 | 0.591739177 | 0.003720571 |
| GPR137B | A_23_P46149 | 0.592900191 | 0.003636814 |
| SLC16A6 | A_23_P152791 | 0.593453522 | 0.003597455 |
| URB1 | A_23_P57293 | 0.593837416 | 0.003570359 |
| MNS1 | A_23_P3302 | 0.594420117 | 0.003529557 |
| HPSE | A_23_P256107 | 0.5944361 | 0.003528443 |
| ARSG | A_23_P4161 | 0.596002524 | 0.00342072 |
| MRS2 | A_23_P111373 | 0.596600427 | 0.003380336 |
| PCNA | A_23_P28886 | 0.597011026 | 0.003352835 |
| ZNF235 | A_23_P208325 | 0.598026478 | 0.003285628 |
| INHBB | A_23_P153958 | 0.599466759 | 0.003192245 |
| LOC391358 | A_24_P280926 | 0.599799991 | 0.003170959 |
| SNRPD1 | A_32_P148672 | 0.600947925 | 0.003098545 |
| POLD3 | A_32_P182439 | 0.601919917 | 0.003038319 |
| TLL1 | A_24_P145163 | 0.603217522 | 0.002959454 |
| PLA2G7 | A_23_P145096 | 0.603361409 | 0.002950816 |
| LOC492303 | A_32_P197620 | 0.603561058 | 0.002938865 |
| SEC23B | A_24_P690924 | 0.604122411 | 0.002905481 |
| YSK4 | A_24_P63078 | 0.60471408 | 0.00287064 |
| C20orf72 | A_24_P254285 | 0.605771428 | 0.002809251 |
| CHERP | A_23_P16139 | 0.605845142 | 0.002805013 |
| MSL3L2 | A_32_P235159 | 0.607408778 | 0.002716368 |
| ZNF606 | A_23_P4628 | 0.608278527 | 0.00266809 |
| GINS2 | A_23_P118246 | 0.608679888 | 0.002646056 |
| ARMC3 | A_23_P86540 | 0.609160511 | 0.002619872 |
| SPATA24 | A_24_P220897 | 0.612076786 | 0.002465629 |
| ROBLD3 | A_23_P103698 | 0.61216448 | 0.002461112 |
| TMEM138 | A_23_P24723 | 0.615150941 | 0.002311392 |
| CD36 | A_23_P111583 | 0.617023537 | 0.002221491 |
| ENPP3 | A_23_P404536 | 0.617915381 | 0.002179723 |
| PARD6B | A_32_P205637 | 0.621363329 | 0.002024423 |
| ZNF613 | A_23_P208172 | 0.621411193 | 0.002022335 |
| ABCC9 | A_23_P368691 | 0.62286446 | 0.001959793 |
| USP21 | A_24_P139620 | 0.623764159 | 0.001921899 |
| CDC73 | A_24_P285179 | 0.623767923 | 0.001921742 |
| C1orf26 | A_23_P96936 | 0.623868463 | 0.001917546 |
| LOC100132351 | A_32_P42797 | 0.624678348 | 0.001884032 |
| TBCE | A_23_P52147 | 0.625849497 | 0.001836444 |
| RIBC1 | A_23_P73667 | 0.626606008 | 0.001806249 |
| CYP4F2 | A_24_P168495 | 0.626622493 | 0.001805596 |
| ZFP112 | A_23_P107724 | 0.627415528 | 0.001774404 |
| GSTM4 | A_24_P396662 | 0.629263427 | 0.001703488 |
| SPAG1 | A_24_P158161 | 0.629544164 | 0.001692927 |
| C1orf131 | A_23_P46095 | 0.630052981 | 0.001673927 |
| FAM183A | A_24_P847326 | 0.630583907 | 0.001654295 |
| C16orf53 | A_24_P22436 | 0.633325261 | 0.001556003 |
| RACGAP1 | A_23_P65110 | 0.63391951 | 0.001535363 |
| KIF18A | A_23_P150667 | 0.635180255 | 0.00149234 |
| C3orf15 | A_24_P160696 | 0.63539265 | 0.001485193 |
| IFT74 | A_23_P255714 | 0.635551638 | 0.001479863 |
| SLC41A1 | A_24_P122732 | 0.635873244 | 0.00146913 |
| PPIL6 | A_23_P111361 | 0.637340001 | 0.001421014 |
| TEKT2 | A_23_P45955 | 0.637444799 | 0.001417628 |
| ASAH1 | A_23_P216325 | 0.640425977 | 0.001324133 |
| NUP85 | A_23_P26777 | 0.640594208 | 0.001319017 |
| C9orf6 | A_23_P216568 | 0.640745853 | 0.00131442 |
| RAB42 | A_23_P434919 | 0.640802043 | 0.00131272 |
| LSM6 | A_23_P259451 | 0.646382333 | 0.001152935 |
| PLIN1 | A_23_P26154 | 0.647636163 | 0.001119407 |
| CHCHD5 | A_23_P154282 | 0.647848701 | 0.001113807 |
| CKS1B | A_32_P206698 | 0.648383627 | 0.001099817 |
| ERCC4 | A_32_P65061 | 0.65102427 | 0.001032929 |
| ZNF227 | A_23_P371011 | 0.65244503 | 0.000998397 |
| KLKBL4 | A_23_P88776 | 0.653794307 | 0.000966519 |
| CSNK2A1 | A_24_P936444 | 0.656761755 | 0.000899449 |
| GNPAT | A_23_P85777 | 0.663645018 | 0.000758988 |
| EP300 | A_23_P40693 | 0.663649419 | 0.000758904 |
| VPS37A | A_24_P941649 | 0.664828323 | 0.000736838 |
| CHCHD5 | A_24_P379727 | 0.666664886 | 0.000703555 |
| ZNF615 | A_23_P67072 | 0.671104272 | 0.000628368 |
| ZNF234 | A_24_P924329 | 0.675634205 | 0.000558848 |
| SCYL3 | A_23_P74320 | 0.676143053 | 0.000551468 |
| HOXA10 | A_24_P77904 | 0.681394454 | 0.000480052 |
| HOXA7 | A_23_P70968 | 0.685697492 | 0.000427599 |
| LOC439911 | A_24_P633543 | 0.686292294 | 0.000420751 |
| DHX16 | A_23_P168062 | 0.688325931 | 0.000398046 |
| HOXA10 | A_23_P253368 | 0.695927842 | 0.000322243 |
| TAF1A | A_23_P74663 | 0.69744914 | 0.00030867 |
| PEBP4 | A_23_P157636 | 0.700899813 | 0.000279695 |
| HOXA11 | A_23_P42706 | 0.709030507 | 0.000220527 |
| TAF6L | A_23_P47541 | 0.712225504 | 0.000200431 |
| GSTM4 | A_24_P396660 | 0.720094647 | 0.000157547 |
| ZNF432 | A_23_P208210 | 0.722276621 | 0.000147167 |
| DPM3 | A_24_P179925 | 0.724812097 | 0.000135853 |
| GSTM4 | A_23_P217917 | 0.726125218 | 0.000130296 |
| HOXA11AS | A_24_P280983 | 0.833178656 | 1.48E-06 |
